# International cross-sectional survey on current and updated definitions of intra-abdominal hypertension and abdominal compartment syndrome

**DOI:** 10.1186/s13017-024-00564-5

**Published:** 2024-11-29

**Authors:** Prashant Nasa, Robert D. Wise, Marije Smit, Stefan Acosta, Scott D’Amours, William Beaubien–Souligny, Zsolt Bodnar, Federico Coccolini, Neha S. Dangayach, Wojciech Dabrowski, Juan Duchesne, Janeth C. Ejike, Goran Augustin, Bart De Keulenaer, Andrew W. Kirkpatrick, Ashish K. Khanna, Edward Kimball, Abhilash Koratala, Rosemary K. Lee, Ari Leppaniemi, Edgar V. Lerma, Valerie Marmolejo, Alejando Meraz–Munoz, Sheila N. Myatra, Daniel Niven, Claudia Olvera, Carlos Ordoñez, Clayton Petro, Bruno M. Pereira, Claudio Ronco, Adrian Regli, Derek J. Roberts, Philippe Rola, Michael Rosen, Gentle S. Shrestha, Michael Sugrue, Juan Carlos Q. Velez, Ron Wald, Jan De Waele, Annika Reintam Blaser, Manu L. N. G. Malbrain

**Affiliations:** 1 Department of Anaesthesia and Critical Care Medicine, The Royal Wolverhampton NHS Trust, New Cross Hospital, Wolverhampton, WV10 0QP UK; 2 Faculty of Medicine and Pharmacy, Vrije Universiteit Brussel (VUB), 1050 Brussels, Belgium; 3 Discipline of Anesthesiology and Critical Care, School of Clinical Medicine, University of KwaZulu–Natal, Durban, 4001 South Africa; 4 Adult Intensive Care, John Radcliffe Hospital, Oxford University Hospitals NHS Foundation Trust, Oxford, OX3 9DU UK; 5 Department of Critical Care, University Medical Center Groningen, University of Groningen, Groningen, The Netherlands; 6 Department of Clinical Sciences, Malmö, Lund University, Malmö, Sweden; 7 Trauma and Acute Care Surgery Unit, Liverpool Hospital, Sydney, Australia; 8 Department of Medicine, Nephrology Division, Centre Hospitalier de L’Université de Montréal, Université de Montréal, Montreal, Canada; 9 Department of Surgery, Letterkenny University Hospital, Donegal, Ireland; 10 General, Emergency and Trauma Surgery Department, Pisa University Hospital, Pisa, Italy; 11 Department of Neurosurgery, Icahn School of Medicine at Mount Sinai, New York, NY USA; 12 First Department of Anesthesiology and Intensive Therapy, Medical University of Lublin, Lublin, Poland; 13 Division Chief Trauma/Acute Care and Critical Care Department of Surgery, Tulane University, New Orleans, LA USA; 14 Department of Pediatrics, Downey Medical Center, Southern California Permanente Medical Group, 9333 Imperial Highway, Downey, CA 90242 USA; 15 Kaiser Permanente Bernard J. Tyson School of Medicine, 98 S. Los Robles, 2nd Floor, Pasadena, CA 91101 USA; 16 Department of Surgery, University Hospital Centre Zagreb, Zagreb, Croatia; 17 Department of Intensive Care, Fiona Stanley Hospital, Murdoch, Australia; 18 Department of Surgery, The University of Western Australia, Perth, WA Australia; 19 Department of Surgery and Critical Care Medicine, Regional Trauma Services Foothills Medical Centre, Calgary, AB T2N 2T9 Canada; 20 Department of Anesthesiology, Section On Critical Care Medicine, Wake Forest School of Medicine, Atrium Health Wake Forest Baptist Medical Center, Winston–Salem, NC USA; 21 Outcomes Research Consortium, Houston, TX USA; 22 Department of Surgery, University of Utah, 50 N Medical Drive, Salt Lake City, UT USA; 23 Division of Nephrology Froedtert & Medical College of Wisconsin, Milwaukee, WI USA; 24 Baptist Health South Florida, Coral Gables, Florida USA; 25 Department of Abdominal Surgery, Meilahti Hospital, University of Helsinki, Haartmaninkatu 4, PO Box 340, 00029 Helsinki, Finland; 26 Department of Medicine, Advocate Christ Medical Center, University of Illinois at Chicago, Oak Lawn, IL USA; 27 DPM, MS, Medical Writer, Scriptum Medica, Washington, USA; 28 Division of Nephrology, St. Boniface Hospital and The University of Manitoba, Winnipeg, MB Canada; 29 Department of Anesthesiology, Critical Care and Pain, Tata Memorial Hospital, Homi Bhabha National Institute, Mumbai, India; 30 Departments of Critical Care Medicine and Community Health Sciences, Cumming School of Medicine, University of Calgary, Calgary, AB Canada; 31 The American British Cowdray Medical Center, Universidad Anahuac, Mexico City, Mexico; 32 Division of Trauma and Acute Care Surgery, Department of Surgery, Fundación Valle del Lili, Cra 98 No. 18–49, 760032 Cali, Colombia; 33 Sección de Cirugía de Trauma y Emergencias, Universidad del Valle - Hospital Universitario del Valle, Cl 5 No. 36–08, 760032 Cali, Colombia; 34 Department of General Surgery, Center for Abdominal Core Health, Cleveland Clinic, Cleveland, OH USA; 35 University of Vassouras, Rio de Janeiro, Brazil; 36 Department of Nephrology and the International Renal Research Institute (IRRIV), San Bortolo Hospital, Vicenza, Italy; 37 Department of Intensive Care, Fiona Stanley Hospital, Perth, WA Australia; 38 Medical School, The Notre Dame University, Fremantle, WA Australia; 39 Medical School, The University of Western Australia, Perth, WA Australia; 40 Departments of Surgery and Community Health Sciences, University of Calgary, Calgary, AB T2N 5A1 Canada; 41 Intensive Care, Santa Cabrini Hospital, Montreal, QC Canada; 42 Department of Critical Care Medicine, Tribhuvan University Teaching Hospital, Maharajgunj, Kathmandu, Nepal; 43 Letterkenny University Hospital, Donegal, Ireland; 44 Ochsner Medical Center, New Orleans, LA USA; 45 Division of Nephrology, Department of Medicine, St. Michael’s Hospital, Toronto, Canada; 46 Department of Nephrology and Hypertension, Tel Aviv Medical Center, Tel Aviv, Israel; 47 Department of Intensive Care Medicine, Ghent University Hospital, Ghent, Belgium; 48 Department of Internal Medicine and Pediatrics, Faculty of Medicine and Health Sciences, Ghent University, Ghent, Belgium; 49 Clinic of Anesthesiology and Intensive Care, University of Tartu, Puusepa 8, 51014 Tartu, Estonia; 50 Department of Intensive Care Medicine, Lucerne Cantonal Hospital, Lucerne, Switzerland; 51 Medical Data Management, Medaman, Geel, Belgium; 52 International Fluid Academy, Lovenjoel, Belgium; 53 General Surgery Residency Program, Santa Casa de Campinas, Rio de Janeiro, Brazil; 54 The University of New South Wales- South West Clinical School, Sydney, Australia; 55 University of Padova, Padua, Italy

**Keywords:** Abdominal pressure, Abdominal hypertension, Abdominal compartment syndrome, Definitions, Pathophysiology, Management, Survey

## Abstract

**Background:**

The Abdominal Compartment Society (WSACS) established consensus definitions and recommendations for the management of intra-abdominal hypertension (IAH) and abdominal compartment syndrome (ACS) in 2006, and they were last updated in 2013. The WSACS conducted an international survey between 2022 and 2023 to seek the agreement of healthcare practitioners (HCPs) worldwide on current and new candidate statements that may be used for future guidelines.

**Methods:**

A self-administered, online cross-sectional survey was conducted under the auspices of the WSACS to assess the level of agreement among HCPs over current and new candidate statements. The survey, distributed electronically worldwide, collected agreement or disagreement with statements on the measurement of intra-abdominal pressure (IAP), pathophysiology, definitions, and management of IAH/ACS. Statistical analysis assessed agreement levels, expressed in percentages, on statements among respondents, and comparisons between groups were performed according to the respondent’s education status, base specialty, duration of work experience, role (intensivist vs non-intensivist) and involvement in previous guidelines. Agreement was considered to be reached when 80% or more of the respondents agreed with a particular statement.

**Results:**

A total of 1042 respondents from 102 countries, predominantly physicians (73%), of whom 48% were intensivists, participated. Only 59% of HCPs were aware of the 2013 WSACS guidelines, and 41% incorporated them into practice. Despite agreement in most statements, significant variability existed. Notably, agreement was not reached on four new candidate statements: “normal intra-abdominal pressure (IAP) is 10 mmHg in critically ill adults” (77%), “clinical assessment and estimation of IAP is inaccurate” (65.2%), “intragastric can be an alternative to the intravesical route for IAP measurement” (70.4%), and “measurement of IAP should be repeated in the resting position after measurement in a supine position” (71.9%). The survey elucidated nuances in clinical practice and highlighted areas for further education and standardization.

**Conclusion:**

More than ten years after the last published guidelines, this worldwide cross-sectional survey collected feedback and evaluated the level of agreement with current recommendations and new candidate statements. This will inform the consensus process for future guideline development.

**Supplementary Information:**

The online version contains supplementary material available at 10.1186/s13017-024-00564-5.

## Introduction

The World Society of the Abdominal Compartment Syndrome, founded in 2004, was renamed the Abdominal Compartment Society (WSACS; www.wsacs.org and https://wsacs.mn.co) and developed consensus definitions and recommendations for the management of intra-abdominal hypertension (IAH) and abdominal compartment syndrome (ACS), which were last updated in 2013 [1–3]. In the last decade, considerable progress has been made toward a better understanding of the pathophysiology, accurate diagnosis and management of IAH and ACS.

The 2017 knowledge and awareness surveys on intra-abdominal pressure (IAP), IAH, ACS, and WSACS guidelines revealed an overall improvement in healthcare practitioner (HCP) awareness of WCACS guidelines (60.2% vs 28.4%, *p* < 0.01) from the previous 2007 international survey [4, 5]. However, the level of awareness of the WSACS guidelines remained low (48% vs 42.7%, *p* < 0.01), with 18% of respondents never measuring IAP and 39% relying on clinical examination to diagnose IAH. Another recent survey in neonatal and pediatric intensive care units (ICUs) revealed the scope of improvement in awareness and knowledge among clinicians and the need to develop pediatric-specific diagnostic algorithms for IAH and ACS [6, 7].

Despite, the 2013 WSACS guidelines is a comprehensive document, it remains to be established whether global HCPs agree with the definitions and recommendations or whether additional definitions and/or management approaches are needed. In addition, the current guidelines lack recommendations for IAP measurement in patients with an elevated head-of-bed (HOB) position [8, 9], in patients who are awake and spontaneously breathing, or who receive noninvasive ventilation (NIV) [10]. Additionally, the recommendations are unclear for continuous vs intermittent IAP measurement [11, 12] or measurement techniques in patients with open abdomen management using vacuum-assisted closure (VAC) or negative pressure wound therapy (NPWT) [13, 14].

Thus, the WSACS collaborated with researchers internationally in preparation for the revision of the guidelines**.** An international cross-sectional survey was conducted among HCPs worldwide to determine the level of agreement and feedback on the current guidelines and new candidate statements for a future set of revised consensus guidelines on IAH and ACS.

## Methods

### Design

We distributed an electronic, international survey under the aegis of the WSACS between March 2022 and July 2023 (Supplement page 1).

The WSACS is an international, integrated, not-for-profit organization under Belgian law that aims to promote the education of medical or paramedical personnel on IAH, ACS, and all aspects of caring for critically ill patients with acute abdominal problems, as well as to foster scientific research in this area. The survey questionnaire was hosted as a live document on SurveyMonkey®, made available to responders via a web link, was disseminated through the website (www.wsacs.org), social media channels of WSACS and the International Fluid Academy (IFA), and e-mail communication to HCPs registered as members with the WSACS or IFA under the General Data Protection Regulation (GDPR) law. The WSACS Executive Board approved the study, and the STROBE guidelines were followed to report the findings of this cross-sectional survey (Supplement pages 2 to 3).

### Study population

The survey was open to all HCPs interested in the research or management of patients with IAH or ACS.

### Survey questionnaire

The steering committee (PN, MS, and MLNGM) prepared the survey. The survey was divided into three sections: respondent characteristics; commentary on statements; and awareness and advocacy of WSACS missions. The demographic details collected included education status, medical speciality, country, and duration of work experience. The statements of the survey included recommendations of the 2013 WSACS guidelines and candidate statements based on the electronic feedback in 2019 and later on repeated after COVID in 2022, that would that would be tested by a formal consensus method among the expert panel (co-authors) planning to revise the guidelines. The respondents agreed or disagreed with the statements, and optional feedback was collected for each statement. A positive agreement was defined when 80% or more of the respondents agreed with a statement. The final section collected information on awareness of the WSACS guidelines, interest in advocacy and guideline processes and the future of WSACS (Supplement pages 4 to 15). The steering group pilot tested the survey to assess the clarity and brevity of the statements.

Ethics approval was waived because of the survey’s online format, and only healthcare professionals participated. The survey and analysis were performed in accordance with the principles outlined in the Declaration of Helsinki. Completion of the survey implied consent for participation, anonymized data processing, and publication of the results. Only deidentified data were used for analysis.

### Statistical analysis

The survey responses are presented as counts (percentages), means (± standard deviations) and medians (interquartile ranges). A Kolmogorov-Smirnov test was used to determine whether the data were normally distributed. Quantitative variables were compared between the study groups via the Mann-Whitney U test and Kruskal-Wallis test for nonparametric data. The chi-square (χ^2^) test was performed to compare categorical data. The level of agreement, expressed in percentages, on statements was compared between groups according to the respondent’s education status, medical specialty, duration of professional experience, and whether they were based on collaboration or not in previous guidelines. The mean score for each domain was also compared on the basis of these variables. All data analyses were performed via Stata version 12.1 (StataCorp LLC, 4905 Lakeway Drive, College Station, TX, USA).

## Results

**Fig. 1:**
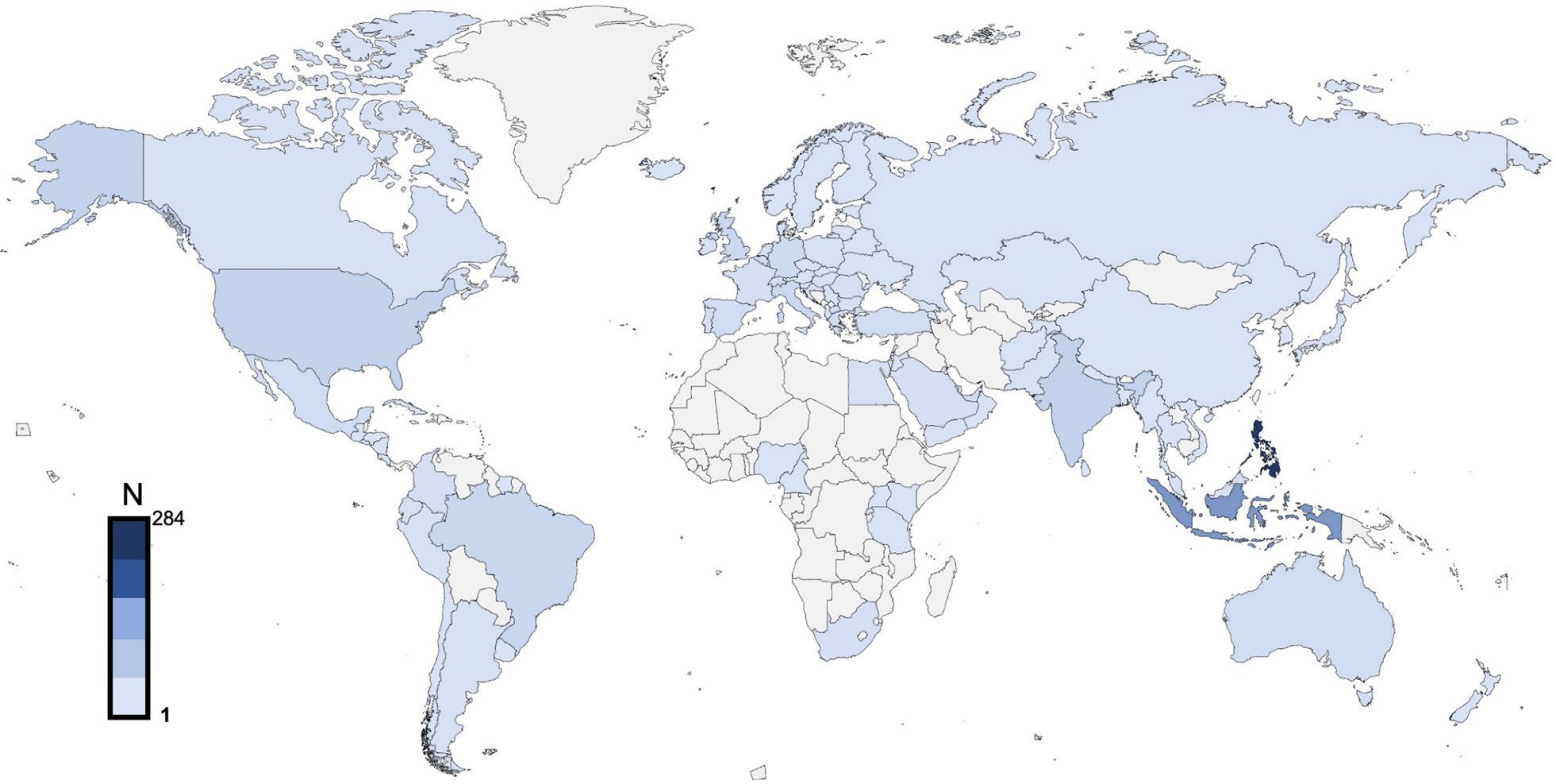
Geographical representation of the respondents of the cross-sectional survey

**Fig. 2:**
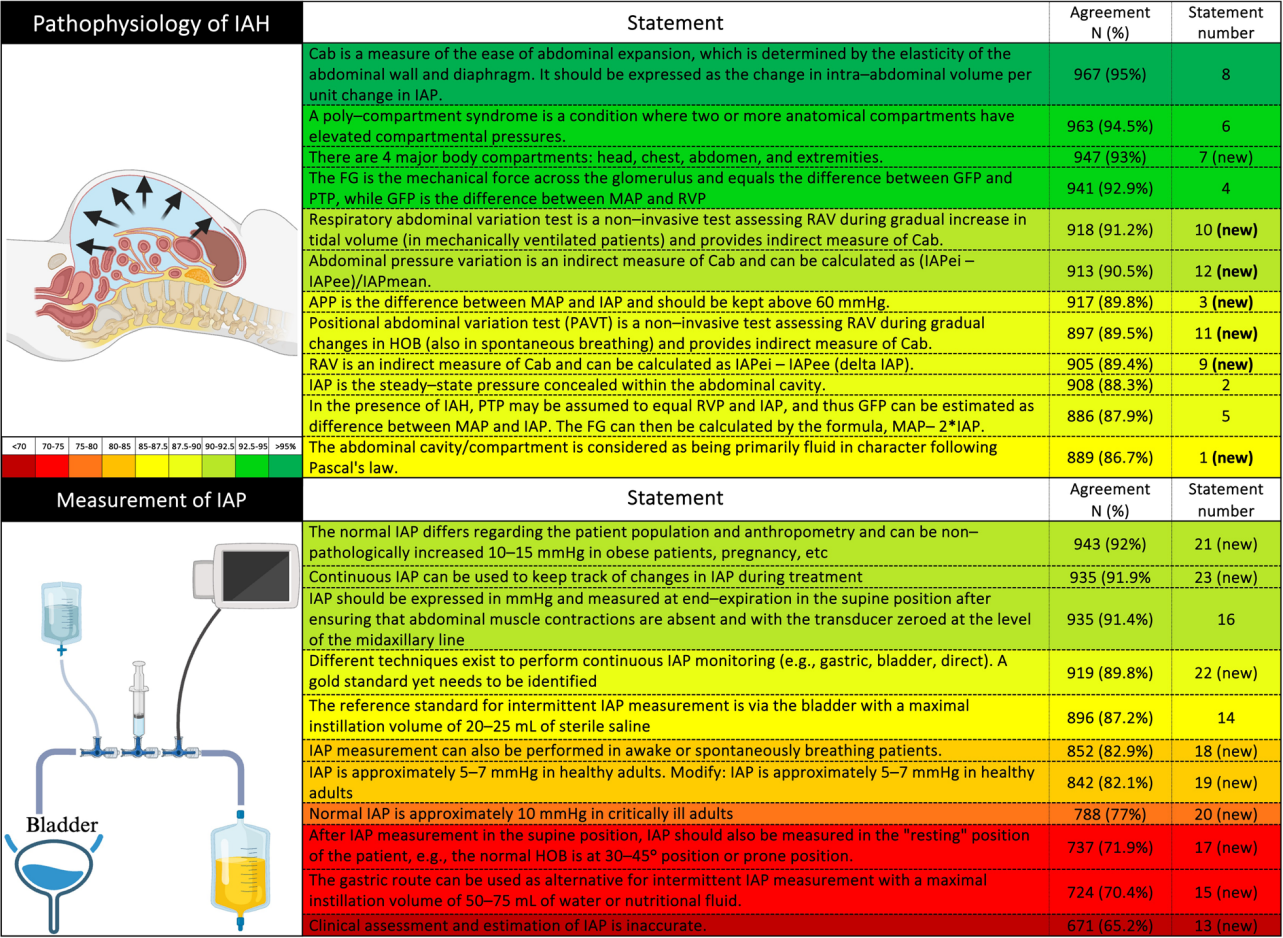
Respondents’ agreement with statements related to the pathophysiology of intra-abdominal hypertension and measurement of intraabdominal pressure. MAP: mean arterial pressure, IAP: intrabdominal pressure, FG: filtration gradient, GFP: glomerular filtration pressure, APP: abdominal perfusion pressure, PTP: proximal tubular pressure, Cab: abdominal compliance, RVP: renal venous pressure, IAPei: IAP end-inspiratory, IAPee: IAP end-expiratory, HOB: head-of-bed, RAV: respiratory-abdominal variation, (new) are candidate statements for future revision of guidelines

**Fig. 3:**
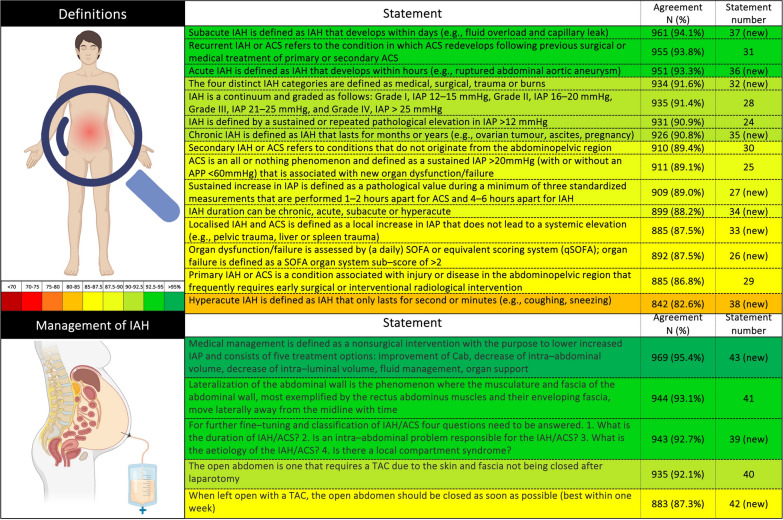
Respondents’ agreement with statements related to the definition and management of intra-abdominal hypertension and abdominal compartment syndrome. ACS: abdominal compartment syndrome, IAH: intrabdominal hypertension, IAP: intrabdominal pressure, APP: abdominal perfusion pressure, SOFA: sequential failure organ assessment, qSOFA: quick SOFA, TAC: temporary abdominal closure, (new) in the parenthesis are candidate statements for future revision of guidelines

The survey consisted of 43 statements, which were classified into broader domains of pathophysiology, definitions, measurement of IAP and management of IAH/ACS. The survey was completed by 1042 respondents from 102 countries (Fig. 1), with a median work experience of 10 (3-20) years. Among those who responded, 737 (71%) were physicians, including 48 (5%) trainees, and 486 (47%) were intensivists. The base specialties of the responding physicians were anesthesia (377, 51%), internal medicine (137, 18%), surgery (139, 19%), pediatrics (55, 7%), and emergency medicine (47, 6%). Many (552, 53%) respondents were aware of WSACS, and 269 (26%) were long-term members of WSACS. Four hundred twenty-eight (41%) respondents were unaware of the WSACS 2013 guidelines, the majority (346, 81%) of whom belong to low- and middle-income countries. Only 41% of HCPs were using them in their clinical practice at the time of the survey. Most statements reached the predefined positive agreement level (≥ 80%) (Figs. 2 and 3). Positive agreement was not achieved for the following new candidate statements: “normal IAP is 10 mmHg in critically ill adults” (77%), “clinical assessment and estimation of IAP is inaccurate” (65.2%), “intragastric route is an alternative to intravesical IAP measurement” (70.4%), and “measurement of IAP at the resting position should be repeated in the supine position” (71.9%). A comparison between groups on the basis of education status, base specialty, duration of professional experience, intensivist vs. non-intensivist, and collaborators in previous guidelines is presented in Supplement pages 16 to 26. The relevant comments from the respondents on the statements are outlined in the Supplement pages 26 to 56.

### Future of WSACS

**Fig. 4:**
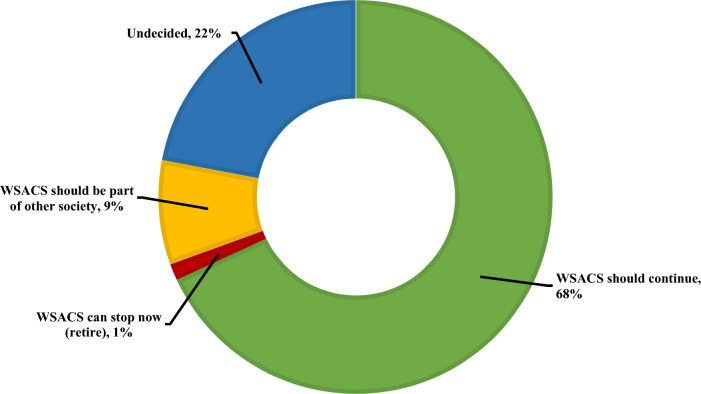
Distribution of respondents’ opinions on the future role of the Abdominal Compartment Society, WSACS: Abdominal Compartment Society

*A total of* 1039 (99%) respondents provided feedback on the future of WSACS, and a clear majority (n = 700, 67.4%) felt that society should continue its endeavors to foster education and training and promote research on IAH and ACS (Fig. 4). A minority of respondents felt that WSACS should be part of another society (89, 8.5%).

## Discussion

There was positive agreement among HCPs worldwide over 2013 WSACS recommendations and draft statements on the pathophysiology, definition, measurement of IAP, and management of IAH and ACS. The new candidate statements for which agreement was less broad included: (1) A normal IAP of 10 mmHg in critically ill patients, (2) The accuracy of the clinical assessment and estimation of the IAP, (3) The use of the intragastric route as an alternative to the intravesical route for IAP measurement, and (4) Patient positioning for measurement of the IAP. The results of this survey and the comments will inform the development of future WSACS consensus guidelines.

### IAP and the pathophysiology of IAH

The abdominal cavity can be assumed to be an enclosed space surrounded by rigid bones (lower ribs, costal arch, spine and pelvis) and a partially stretchable abdominal wall [15]. For the measurement of IAP, the abdominal cavity and its contents can be considered relatively noncompressible and fluid in character, to which Pascal’s law can be applied. Pascal’s law states that pressure change at any point of an enclosed incompressible fluid compartment is equally transmitted to every other point and to the walls of the compartment. Hence, pressure measured at one point is representative of pressure throughout the abdominal cavity, and the IAP can be estimated at various locations, including the bladder (most commonly), stomach, rectum, uterus or inferior vena cava [7, 16, 17]. This oversimplification was challenged by a few respondents, who argued that tissues of different densities, such as gas and solid (abdominal viscera, stools, etc.), are common contents of the abdomen. Some respondents even reported that the IAP is a steady-state pressure within the abdominal cavity and pointed to physiological variations during respiration or positive pressure ventilation, along with routine changes in the abdominal contents and hydration status.

Abdominal wall compliance (C_ab_) is a surrogate for abdominal wall expansion and is determined mainly by the abdominal wall muscles and, to a lesser extent, diaphragm elasticity. The C_ab_ is measured by the ratio of the change in intraabdominal volume (∆IAV) to the change in IAP at the end of expiration (∆IAP_ee_) at a given time point. For example, if for a 1000 mL increase in IAV, IAP_ee_ would increase from 10 to 15 mmHg, the C_ab_ would then be equal to 1000/(15–10), or thus 200 ml/mmHg. As ∆IAV is usually unknown, tidal volume (V_T_) excursions in mL can be used instead, and ∆IAP can be simplified and further calculated by the difference between IAP end-inspiration (IAP_ei_) and IAP_ee_ [18–20]. The relationship between the IAV and IAP (pressure-volume curve) of C_ab_ is curvilinear, with an initial phase being linear [21]. On the other hand, at higher grades of IAH, minor changes in the IAV produce an exponential increase in the IAP, and vice versa. The initial position on the pressure-volume curve is important for determining the actual C_ab_ [22, 23]. Abdominal pressure variation (APV) is a noninvasive surrogate of C_ab_ and is calculated as a percentage of the ∆IAP to the mean IAP. There is an inverse relationship between APV and C_ab_. The respiratory abdominal variation test (RAVT) measures C_ab_ in patients on invasive mechanical ventilation via the following equation: tidal volume change (∆V_T_)/∆IAP_ei_. An incremental ∆V_T_ (e.g., from 4 over 6 to 8 ml/kg) will only increase IAP_ei_ [18, 24]. In spontaneously breathing patients, APV produced by gradual changes in the HOB can be used for C_ab_ measurement (positional abdominal variation test) [18, 25]. The emergence of continuous IAP monitoring techniques will provide further insights into heart–lung-abdominal interactions. Overall, comments received from respondents for these equations highlight the need for further clarification and evidence regarding the clinical utility, validation and measurement methodology of abdominal compliance.

There are four major compartments in the body: the head, thorax, abdomen, and extremities. The pathological rise in pressure in one compartment may lead to organ dysfunction in other compartments because of intercompartmental and organ-organ crosstalk interactions, which is referred to as poly-compartment syndrome (PCS) [26]. The comments about the presence of additional compartments, such as the retroperitoneum, pelvis, and omentum highlight the complexity and diversity of anatomical compartments that warrant consideration beyond the four major compartments initially proposed.

The percentage pressure transmission from the thorax to the abdomen is called the thoracoabdominal index, and from the abdomen to the thorax, the abdominothoracic index of transmission, which is, on average, approximately 50% [27–29]. Rapid-onset multiorgan dysfunction may result from PCS and is associated with high morbidity and mortality [30]. The abdominal perfusion pressure (APP) is calculated as the difference between the MAP and the IAP and is a marker of visceral perfusion and a better predictor of outcomes in critically ill patients than the IAP alone [31, 32]. There is some evidence for the superiority of APP- over MAP-targeted resuscitation in patients with sepsis to prevent a decline in the glomerular filtration rate (GFR) [33, 34]. However, the respondents expressed uncertainty and a lack of evidence for the target of 60 mmHg for the APP and suggested a more individualized approach.

Acute kidney injury (AKI) is a consistent manifestation of IAH/ACS [34–36]. IAH reduces renal perfusion pressure (RPP) and the filtration gradient (FG). In normal individuals, FG is calculated as the difference between the glomerulus filtration pressure (GFP) and proximal tubular pressure (PTP). However, in the presence of IAH, the GFP is dependent on the difference between the mean arterial pressure (MAP) and the IAP, and the PTP is approximated as the IAP. Thus, the equation of FG can be amended as the difference between MAP and two times the IAP, illustrating the greater impact of IAP on FG [2, 15]. Other proposed formulas for RPP include MAP–IAP–central venous pressure (CVP), and in mechanically ventilated patients, MAP–IAP–CVP–P_mean_ (where P_mean_ is the mean alveolar pressure) [37]. IAH may cause or exacerbate AKI, and new-onset oliguria/anuria may increase the risk of ACS in patients with IAH [34]. Future guidelines should include the dynamics and respiratory variations of IAP measurements and address the importance of C_ab_, organ-organ interactions (PCS), the role of perfusion pressures (APP, RPP) and venous congestion [38]. Few respondents expressed concern and suggested further research and evidence to support the validity and usefulness of the formula in clinical practice, considering the complexity of equations.

### Measurement of the IAP

Some studies have shown a lower accuracy and sensitivity of the clinical estimation of the IAP than of the quantitative measurement of the IAP [39, 40]. However, in this survey, nearly one-third of the respondents agreed with the statement that the clinical estimation of IAP is accurate. The respondents commented that clinical assessment may provide useful information in some instances, but direct measurement of the IAP remains the gold standard for accuracy. HCPs with less than ten years of clinical experience, nonintensivists and physicians other than those from internal medicine and surgery were in favor of the clinical estimation of IAP. Similar findings were reported in other cross-sectional surveys on knowledge and awareness of IAH and ACS [4, 5, 41]. In our opinion, clinical examination not only underestimates the IAP but also, more importantly, delays the timely management of IAH and ACS [39]. Our results emphasize the need for continuous education, advocacy and awareness about IAH/ACS among HCPs.

IAP measurement through the intravesical route using an instillation volume of 20-25 ml of sterile saline is widely used and is currently considered a reference standard [3]. Despite the agreement, the respondents expressed divergent opinions on the optimal volume of saline. The intragastric route using a 50-75 ml instillation volume has been suggested as a valid alternative for IAP measurement [42–44]. However, the statement failed to reach the desired agreement because the respondents emphasized the need for further validation, clear guidelines, and evidence supporting its use. Bladder or intragastric routes are traditionally the preferred techniques for continuously monitoring IAP [43]. The WSACS guidelines recommend intermittent IAP measurement every 4-6 h in those with suspected or confirmed IAH or ACS. Recently, newer techniques of continuous IAP measurement have been tested with conflicting results [45, 46]. However, some recent results from in vitro, animal, and first-in-human validation with TraumaGuard and Serenno devices seem promising [47, 48]. Despite the use of different methods for the continuous measurement of the IAP, the gold standard has yet to be identified [16, 17]. Continuous intra-abdominal pressure (CIAP) monitoring, which offers numerous benefits that enhance care and outcomes, is essential for managing critically ill patients in the twenty-first century. CIAP allows real-time trend monitoring of the IAP, enabling clinicians to observe dynamic changes and prompt timely interventions to prevent complications. It captures the effects of body position changes on the IAP, aiding patient management. The CIAP assesses treatment effectiveness by showing continuous pressure changes and facilitates the calculation of continuous abdominal perfusion pressure (CAPP), ensuring adequate organ perfusion. It helps calculate the area under the curve (AUC) or the time above a certain IAP threshold (TAT), reflecting the cumulative pressure time burden of elevated pressures and the severity of hypertension. The CIAP also helps identify patients at risk of complications. It provides insights into PCS by monitoring interactions between different body compartments, such as the abdomen, thorax, and brain. The abdominal-thoracic index (ATI) and thoracoabdominal index (TAI) can be monitored to understand intercompartmental pressure transmission, aiding in optimizing mechanical ventilation settings [12]. The IAP should be measured in the supine position at end-expiration, with the transducer zeroed at the midaxillary level [1]. HOB elevation can significantly increase the IAP [16, 49, 50]. Nevertheless, the IAP can be measured in an elevated HOB or prone position, but a consistent body position should be maintained during serial measurements [51]. Many respondents questioned the relevance and implications of measuring the IAP in different positions and suggested standardization of the measurement of IAP, especially, practicality concerns while measuring in prone positioning.

Previous WSACS guidelines recommended that abdominal contractions be absent during IAP measurement [2, 3]. This translates to IAP measurements being more reliable in a completely sedated and mechanically ventilated patient. However, it is a misconception that patients must be fully asleep or under neuromuscular blockers to obtain a correct IAP value or that the IAP is untrustworthy in awake patients or those receiving noninvasive mechanical ventilation [52]. In this survey, a greater proportion of intensivists and those who participated in previous WSACS research or guidelines agreed with the statement that IAP measurements are trustworthy in awake and spontaneously breathing patients. There are a few reservations about its accuracy and interpretability, necessitating careful consideration and further validation.

The baseline IAP varies across individuals, and previous guidelines recommended a baseline IAP of 5-7 mmHg in critically ill adults [3]. Non-intensivists and physicians (internal medicine and surgery) favored a 5-10 mmHg baseline IAP for healthy adults. Researchers have not clearly determined whether healthy adults are obese or pregnant and have suggested a broader range for physiological IAP. Some researchers have proposed modifications to a range of 0-5 mmHg in healthy adults and emphasized the impact of factors such as body mass index (BMI) on IAP.

Considering the impact of disease severity and position and the impact of interventions such as mechanical ventilation on critically ill patients, a higher threshold for normal IAP (~ 10 mmHg) may be more reasonable. However, an agreement regarding the statement could not be reached. There are queries about the evidence supporting this assertion and concerns about defining a *single value* as "normal" for critically ill patients, given the variability in disease and patient characteristics. A few respondents proposed a range of 7-12 mmHg. However, the baseline IAP varies widely, and higher baseline IAP values of 12-14 mmHg have been reported in morbidly obese, obstetric, and liver cirrhosis patients with ascites [52–54].

### Definitions

In critically ill patients, IAH is defined as sustained or recurrent elevation of the IAP equal to or above 12 mmHg [1, 55]. Some respondents suggested defining “sustained” and “repeated” and increasing the threshold to more than 15 mmHg because of factors such as high BMI. Similarly, rather than a specific IAP, respondents suggested focusing on organ dysfunction (not necessarily using SOFA or quick SOFA score) to define ACS. In awake, non-critically ill patients without risk factors for IAH, abdominal muscle activity may transiently increase the IAP to as high as 20 mmHg [49]. Some laboratory data support sustained exposure for 90 min to even slightly elevated IAP, which may increase intestinal permeability and mucosal damage in rats [56, 57]. However, there is a lack of human data to support the fact that transient IAP increases to produce any discernible organ dysfunction. Hence, the diagnosis of IAH/ACS requires a sustained increase in IAP in three or more measurements over 1-2 h apart for ACS and 4-6 h apart for IAH. The respondents suggested the use of a clinical context rather than an arbitrary duration and more frequent or continuous measurements of IAP in ACS patients to determine the frequency of measurement. The correlation between the impact of IAH and severity grade is controversial, and even lower grades may be associated with a negative impact on tissue perfusion and patient outcomes such as length of stay or duration of mechanical ventilation [58]. Thus, the diagnosis of ACS is not dependent solely on the absolute value of the IAP but also on new-onset organ dysfunction/failure [53]. The factors that need to be considered for diagnosis include the technique and context of IAP measurement, baseline IAP, rapid progression, and duration of IAH [7].

IAH is characterized by a continuum from asymptomatic elevation of the IAP to life-threatening organ dysfunction/failure known as ACS, which requires immediate intervention. Depending on the absolute value of the IAP, IAH can be graded as Grade I (IAP 12-15 mmHg), Grade II (IAP 16-20 mmHg), Grade III (IAP 21-25 mmHg), or Grade IV (IAP > 25 mmHg) [1, 7]. Few respondents suggested two grades using only a cutoff of 20 mmHg to simplify and reduce unnecessary complexity.

IAH can be classified on the basis of etiology, acuity of onset, and risk factors. Patients with IAH can be broadly divided into medical, surgical, trauma and burn patients [59, 60]. Although, ACS is nowadays an uncommon diagnosis in critically ill adults (incidence rate of 0.17%), the associated morbidity and mortality is significantly higher compared to patients without ACS. Gastrointestinal and cardiovascular are common etiologies for ACS [61]. IAH (or ACS) can be defined as primary or secondary, respectively, on the basis of whether the origin of the inciting condition or disease is within the abdominopelvic region. Primary IAH caused by abdominal trauma, peritonitis, surgery, intrabdominal masses, or ascites frequently requires radiological or surgical intervention for its management [59]. Whereas, secondary IAH is caused by the systemic causes in the absence of primary intraperitoneal injury or intervention. Recurrent IAH (or ACS) is characterized by a resurgence after the treatment of primary or secondary IAH/ACS and has a worse patient prognosis [62, 63]. Compared with the absolute IAP, the classification based on the acuity of onset as hyperacute, acute, subacute, or chronic is of greater prognostic significance [64]. However, few respondents expressed ambiguity in the current classification, especially in overlapping conditions, retroperitoneum pathology, and definitions of radiological intervention.

Future definitions should establish what is meant by “consecutive” measurements and “sustained” increased IAP, and this should probably not differ between IAH and ACS to avoid confusion, especially in light of new continuous IAP monitoring techniques, as discussed previously. These new monitoring tools also allow us to calculate other derived parameters, such as the area under the curve or the time above a certain threshold. Analogous to increased intracranial pressure, the pressure-time burden is likely more strongly correlated with adverse outcomes than a single increased IAP value [65, 66]. Few respondents argued against localized IAH, as it did not manifest a systemic elevation and suggested revising it to "organ-specific IAH". Future guidelines must better define and classify the different distinct types of IAH and ACS.

### Management of IAH and ACS

The optimal management of patients with IAH/ACS should consider the duration and etiology of IAH/ACS, the presence of an intra-abdominal pathology and/or the development of local compartment syndrome. The respondents asked for clarification on “local” compartment syndrome and proposed other considerations, such as assessing the trajectory or the consequences of the condition and response to previous therapies for IAH/ACS. The duration of IAH (or thus the pressure time burden) rather than the development of IAH was found to be an independent predictor of 60-day mortality in surgical patients [67]. In another prospective study of critically ill surgical patients, a longer duration of IAH was associated with greater serum lactate and organ dysfunction, longer intensive care unit (ICU) and hospital lengths of stay, longer durations of vasopressor and ventilator requirements and even higher 30-day mortality [68]. The etiology of IAH/ACS is another crucial element considered in the classification of IAH/ACS. The etiology of IAH helps determine the type and urgency of treatment. A transient increase in the IAP after elective abdominal hernia repair may be managed conservatively [69–71]. On the other hand, a progressively increasing IAP during resuscitation of a patient in shock, pancreatitis, or peritonitis requires urgent action (e.g., sedation and/or muscle relaxation and/or decompression) [7, 72]. The intra-abdominal cause of IAH/ACS can be an increased intraluminal or extraluminal volume or decreased abdominal wall compliance.

Gastric distention due to gas insufflation during gastroscopy, increased colonic volume with *Clostridium difficile* colitis, or severe constipation are examples of increased intraluminal volume. IAH in such patients requires a high index of clinical suspicion, periodic IAP measurement and urgent imaging. An increased extraluminal volume caused by air, fluid, or blood accumulation is relatively more straightforward to diagnose with bedside ultrasound or CT. The elasticity of the abdominal wall and diaphragm determines the C_ab_. Decreased C_ab_ is associated with altered body habits (e.g., morbid obesity), decreased abdominal wall elasticity (e.g., rectus sheath hematoma, burn eschars, tight bandages or sutures), and increased abdominal wall volume (e.g., capillary leakage in patients with acute pancreatitis, sepsis, burns) [7, 73, 74]. Finally, the urgency of the management of IAH depends on the degree of organ dysfunction or compartment syndrome. The degree and velocity of the increase in IAP determine the timing and extent of intervention.

The medical management of IAH/ACS consists of lowering the intraluminal and extraluminal volume, improving abdominal wall compliance, and supportive management, including organ support and judicious fluid management [75]. Pharmacological (e.g., prokinetics, enemas) and nonpharmacological (e.g., nasogastric, rectal, or endoscopic decompression) methods can be used to reduce the intraluminal volume [3]. Nonsurgical management for lowering extraluminal volume through percutaneous drainage of fluid collection was found to be effective in acute pancreatitis [69], ascites with liver cirrhosis [76], and burn patients [77–79] with ACS. Escharotomy in burn patients, the release of tight sutures/dressing, or a change in body position are a few simple interventions that can rapidly restore C_ab_ [80, 81].

Fluid management is challenging in patients with IAH/ACS. Overzealous fluid resuscitation may contribute to fluid accumulation and secondary IAH [74]. Furthermore, ongoing fluid administration to manage fluid-responsive shock with IAH only improves cardiac output without improving APP and organ perfusion. Notably, inappropriate fluid therapy may lead to an increase in the IAP, which closely correlates with the extracellular water content in critically ill patients and patients undergoing extra-abdominal surgery [81]. Future research is needed to identify the best resuscitation targets and the type, timing and volume of fluids used in patients with IAH [80]. Some respondents suggested a greater focus on medical management, explaining the role of fluid management, including diuresis.

IAH/ACS can impact any organ or present as polycompartment syndrome (PCS). Most patients need organ support, including respiratory and cardiovascular monitoring [82]. Decompressive laparotomy decreases the IAP rapidly and significantly by increasing the intra-abdominal cavity and potentially decreasing the intra-abdominal volume by removing additional pathological volume (e.g., hematoma, ascites, abscess) and is essential for the management of medically refractory ACS [72, 83]. However, primary fascial closure after decompressive laparotomy is not possible in many patients with an open abdomen (OA) [83]. The goals of OA management include the use of an abdominal cover to protect the bowel from injury and contamination, continued/continuous monitoring of the IAP and prevention of IAH recurrence, fluid management (ascites and intravenous fluids), and early abdominal closure [85].

Different techniques for temporary abdominal closure (TAC) have been described. Wittmann patch (WP) and negative pressure wound therapy (NPWT), sometimes combined with mesh-mediated fascial traction in long-term open abdomens, are the most widely used techniques [85]. Commercially available NPWT is recommended as the preferred technique for TAC [86]. However, OAs should be closed as early as possible and preferably within 72 h to one week, or else active consideration for a primary fascial closure at the earliest opportunity [87]. There is a linear relationship between the risk of complications, including enterocutaneous and especially enteroatmospheric fistulae and the duration of OA [86, 87]. These can be especially difficult to manage and are best avoided.

Early return to the operating room, limiting fluid overload through excessive use of crystalloids, and preventing and/or treating IAH, enteric fistulae, and intra-abdominal collections are some of the recommended interventions to facilitate primary fascial closure [87]. Finally, lateralization of the abdominal muscles (caused by adhesions between the intestine and bowel wall) should be prevented [89]. However, the respondents commented on the variations in practices regarding OA, with a focus on fascia closure alone over both the skin and fascia. Most respondents preferred early closure, but a few suggested considerations for resolving the underlying pathology.

Future guidelines should develop a clear medical management algorithm incorporating medical and mechanical interventions to reduce intraluminal and intraabdominal volume to improve C_ab_ and organ perfusion by introducing fluid stewardship [84, 90]. Medical management comes first in patients with secondary IAH/ACS, and surgical decompression can only be used as a last resort. The ideal decompression and TAC technique, as well as the best timing for opening and closing, need to be defined.

### Strengths and limitations

The survey attempts to evaluate the level of agreement and collect feedback on key statements around current and potential future WSACS guidelines among HCPs, encompassing diverse expertise and geographical representation. The results of this survey provide broad feedback to guide an expert panel in revising the current guidelines. The free-text option after each statement provided valuable insights into the current knowledge gaps around the diagnosis and management of IAH and ACS, providing learning points for future education, advocacy, the creation of guidelines and preclinical and clinical research. Finally, a sizable representation of the respondents from low- and middle-income countries (n = 367, 35%) enhanced the generalizability of our survey.

There are several limitations to this survey. The response rate could not be calculated because of the uncertainty in the number of HCPs who may have received an invitation to complete the survey. Although only a quarter of the respondents were members of WSACS, which can be explained by the methods of distribution of the survey, some remarks could have been biased regarding the role of WSACS. Although the respondents had a median work experience of 10 years, the cognizance of definitions and concepts related to IAH and ACS and the level of expertise in managing such patients were not captured. The inherent disadvantages of a cross-sectional survey, such as recollection bias, inability to obtain point prevalence data and failure to track practice trends, are also applicable to this survey. Finally, the unavailability of certain medical devices (e.g., IAP measurement devices), especially in resource-limited settings, may have caused some bias in the level of agreement.

## Conclusion

This international survey generated valuable comments and agreement (>80%) was achieved in 39 out of 43 statements on the measurement of IAP, as well as the pathophysiology, diagnosis, and management of IAH. The results of this survey and the comments will inform the development of future WSACS consensus guidelines.

## Supplementary Information

Additional file 1

## Data Availability

Data is provided within the manuscript or supplementary information files. Further details can be accessed upon request to the corresponding author.
