# Supplementary material for: International cross-sectional survey on current and updated definitions of intra-abdominal hypertension and abdominal compartment syndrome
Source: World J Emerg Surg. 2024 Nov 29;19:39. doi: 10.1186/s13017-024-00564-5 (PMC11605967; doi:10.1186/s13017-024-00564-5)
Supplement: Supplementary file 1 — Additional file 1 [file 13017_2024_564_MOESM1_ESM.pdf]

Supplement

Figure 1: Number of monthly respondents when the survey was active (dotted line represents floating average)

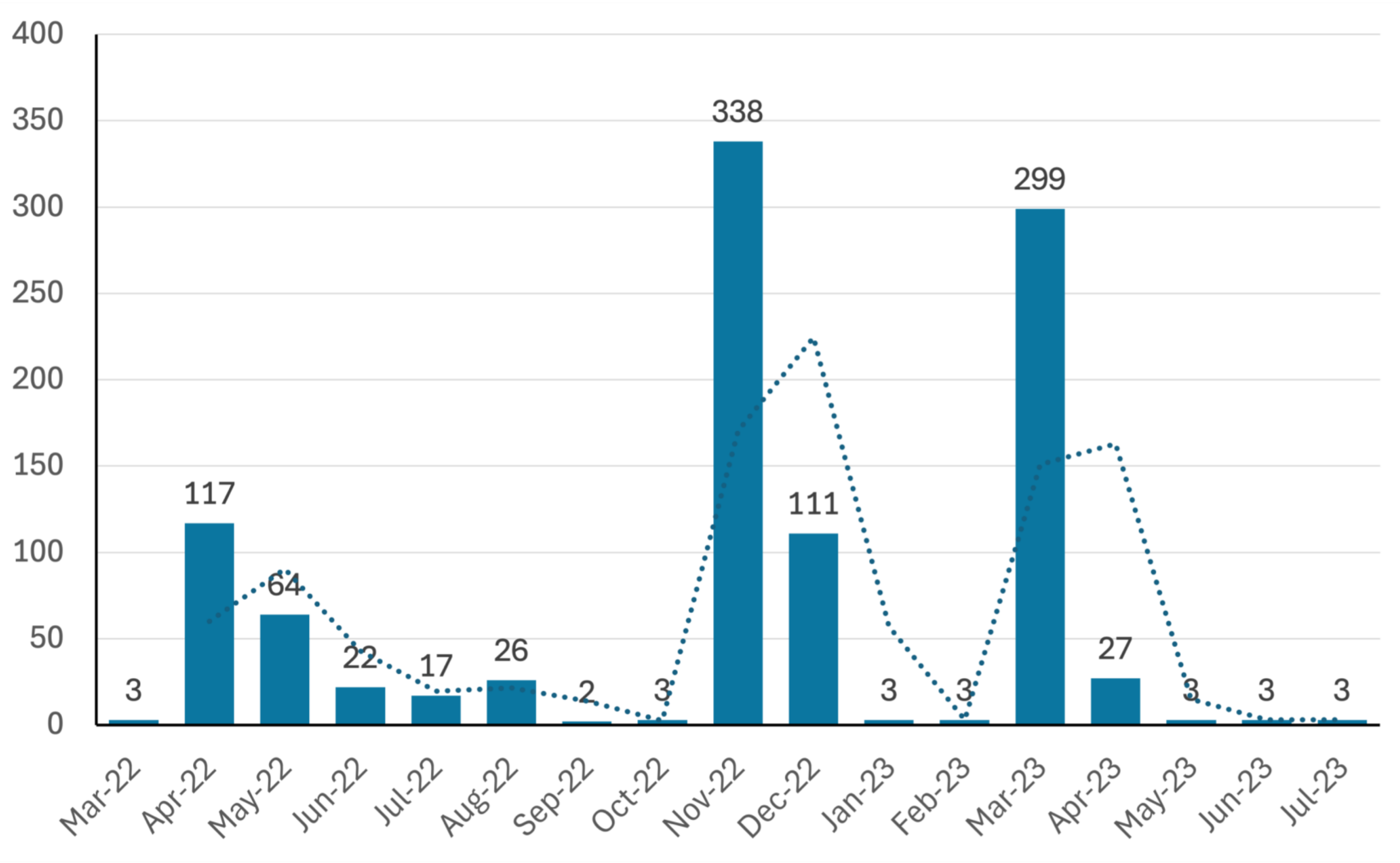

Table 1.

Table 1: Checklist for STROBE statement on reporting of cross-sectional studies

	Item No	Recommendation	Page
**Title and abstract**	1	(*a*) Indicate the study's design with a commonly used term in the title or the abstract	1
		(*b*) Provide in the abstract an informative and balanced summary of what was done and what was found	3,4
**Introduction**			
Background/rationale	2	Explain the scientific background and rationale for the investigation being reported	6,7
Objectives	3	State specific objectives, including any prespecified hypotheses	7
**Methods**			
Study design	4	Present key elements of study design early in the paper	7
Setting	5	Describe the setting, locations, and relevant dates, including periods of recruitment, exposure, follow-up, and data collection	8
Participants	6	(*a*) Give the eligibility criteria, and the sources and methods of selection of participants	8
Variables	7	Clearly define all outcomes, exposures, predictors, potential confounders, and effect modifiers. Give diagnostic criteria, if applicable	8
Data sources/ measurement	8*	For each variable of interest, give sources of data and details of methods of assessment (measurement). Describe comparability of assessment methods if there is more than one group	8,9
Bias	9	Describe any efforts to address potential sources of bias	N/A
Study size	10	Explain how the study size was arrived at	N/A
Quantitative variables	11	Explain how quantitative variables were handled in the analyses. If applicable, describe which groupings were chosen and why	N/A
Statistical methods	12	(*a*) Describe all statistical methods, including those used to control for confounding	8,9
		(*b*) Describe any methods used to examine subgroups and interactions	9
		(*c*) Explain how missing data were addressed	9
		(*d*) If applicable, describe analytical methods taking account of sampling strategy	N/A
		(*e*) Describe any sensitivity analyses	N/A

**Results**			
Participants	13*	(a) Report numbers of individuals at each stage of study—eg numbers potentially eligible, examined for eligibility, confirmed eligible, included in the study, completing follow-up, and analysed	9,10
		(b) Give reasons for non-participation at each stage	-
		(c) Consider use of a flow diagram	Table 1, Supplement Table 2-5
Descriptive data	14*	(a) Give characteristics of study participants (eg demographic, clinical, social) and information on exposures and potential confounders	9,10 Supplement Table 2-5
		(b) Indicate number of participants with missing data for each variable of interest	9, 10
Outcome data	15*	Report numbers of outcome events or summary measures	8, 9
Main results	16	(*a*) Give unadjusted estimates and, if applicable, confounder-adjusted estimates and their precision (eg, 95% confidence interval). Make clear which confounders were adjusted for and why they were included	Figure 2, 3 Appendix commentaries
		(*b*) Report category boundaries when continuous variables were categorized	-
		(*c*) If relevant, consider translating estimates of relative risk into absolute risk for a meaningful time period	NA
Other analyses	17	Report other analyses done—eg analyses of subgroups and interactions, and sensitivity analyses	Supplement Table 2-5
**Discussion**			
Key results	18	Summarise key results with reference to study objectives	10, 11
Limitations	19	Discuss limitations of the study, taking into account sources of potential bias or imprecision. Discuss both direction and magnitude of any potential bias	23, 24
Interpretation	20	Give a cautious overall interpretation of results considering objectives, limitations, multiplicity of analyses, results from similar studies, and other relevant evidence	11-23
Generalisability	21	Discuss the generalisability (external validity) of the study results	23
**Other information**			
Funding	22	Give the source of funding and the role of the funders for the present study and, if applicable, for the original study on which the present article is based	26

WSACS 2022 Consensus Definitions Update

Towards new consensus 2022 definitions on IAH and ACS

PURPOSE:

The first WSACS consensus definitions date back from 2006 with a 2013 update of the World Society of the Abdominal Compartment Syndrome (WSACS) consensus definitions and management statements relating to intra-abdominal hypertension (IAH) and the abdominal compartment syndrome (ACS). After 8 years it is time for a new update, to be released in 2022, which is 20 years after the initial meeting in Sydney.

There is still a lack of clinical awareness and many colleagues do not feel the need for monitoring intra-abdominal pressure (IAP). Furthermore there are still a lot of misconceptions, like IAP is not trustworthy in head-of-bed (HOB) elevation or in patients that are awake, on non-invasive ventilation (NIV) or during spontaneous breathing. Also some believe that during vacuum assisted closure (VAC) or negative pressure wound therapy (NPWT) treatment the IAP can be negative and during open abdomen you cannot develop IAH/ACS so monitoring of IAP is unnecessary, etc...

1. What is your education?

- ◯ Doctor
- ◯ Nurse
- ◯ Doctor in training
- ◯ Nurse in training
- ◯ Industry

Other

[ ]

2. Are you an intensivist?

- ◯ Yes
- ◯ No

3. What is your basic training/speciality (when relevant)?

- ◯ Anaesthesia
- ◯ Internal medicine
- ◯ Surgery
- ◯ Pediatrics
- ◯ Emergency Medicine
- ◯ Not a doctor
- ◯ Other

Other

[ ]

4. How many years of experience do you have (please enter number or enter 0 when still in training)?

5. The abdominal cavity/compartment is considered as being primarily fluid in character following Pascal's law

- ◯ Agree
- ◯ Do not agree

Comments (eg to modify definition)

6. IAP (intra-abdominal pressure) is the steady-state pressure concealed within the abdominal cavity.

- ◯ Agree
- ◯ Do not agree

Comments (eg to modify definition)

7. Clinical assessment and estimation of IAP is inaccurate.

- ◯ Agree
- ◯ Do not agree

Comments (eg to modify definition)

8. The reference standard for intermittent IAP measurement is via the bladder with a maximal instillation volume of 20-25 mL of sterile saline.

- ◯ Agree
- ◯ Do not agree

Comments (eg to modify definition)

9. The gastric route can be used as alternative for intermittent IAP measurement with a maximal instillation volume of 50-75 mL of water or nutritional fluid.

- ◯ Agree
- ◯ Do not agree

Comments (eg to modify definition)

10. IAP should be expressed in mmHg and measured at end-expiration in the supine position after ensuring that abdominal muscle contractions are absent and with the transducer zeroed at the level of the midaxillary line (The conversion factor from mmHg to cmH2O is 1.36 and conversely, from cmH2O to mmHg it is 0.74).

- ◯ Agree
- ◯ Do not agree

Comments (eg to modify definition)

11. After IAP measurement in the supine position, IAP should also be measured in the "resting" position of the patient, eg the normal HOB (head of bed) 30-45° position or prone position

- ◯ Agree
- ◯ Do not agree

Comments (eg to modify definition)

12. IAP measurement can also be performed in awake or spontaneously breathing patients

- ◯ Agree
- ◯ Do not agree

Comments (eg to modify definition)

13. Normal IAP is approximately 5-7 mmHg in critically ill adults. => suggestion to modify as follows: IAP is approximately 5-7 mmHg in healthy adults.

- ◯ Agree
- ◯ Do not agree

Comments (eg to modify definition)

14. Normal IAP is approximately 10 mmHg in critically ill adults.

- ◯ Agree
- ◯ Do not agree

Comments (eg to modify definition)

15. The normal IAP differs regarding the patient population and anthropometry and can be non-pathologically increased 10-15 mmHg in obese patients, pregnancy,...

- ◯ Agree
- ◯ Do not agree

Comments (eg to modify definition)

16. IAH (intra-abdominal hypertension) is defined by a sustained or repeated pathological elevation in IAP > 12 mmHg.

- ◯ Agree
- ◯ Do not agree

Comments (eg to modify definition)

17. ACS (abdominal compartment syndrome) is an all or nothing phenomenon and defined as a sustained IAP>20mmHg (with or without an APP, abdominal perfusion pressure < 60mmHg) that is associated with new organ dysfunction/failure.

- ◯ Agree
- ◯ Do not agree

Comments (eg to modify definition)

18. Organ dysfunction/failure is assessed by (a daily) SOFA (sequential organ failure assessment) or equivalent scoring system (qSOFA); organ failure is defined as a SOFA organ system subscore of >2)

- ◯ Agree
- ◯ Do not agree

Comments (eg to modify definition)

19. Sustained increase in IAP is defined as a pathological value during a minimum of three standardized measurements that are performed 1-2 hours apart for ACS and 4-6 hours apart for IAH

- Agree
- Do not agree

Comments (eg to modify definition)

20. IAH is a continuum and graded as follows: Grade I, IAP 12-15 mmHg, Grade II, IAP 16-20 mmHg, Grade III, IAP 21-25 mmHg, Grade IV, IAP > 25 mmHg

- Agree
- Do not agree

Comments (eg to modify definition)

21. Primary IAH or ACS is a condition associated with injury or disease in the abdominopelvic region that frequently requires early surgical or interventional radiological intervention.

- Agree
- Do not agree

Comments (eg to modify definition)

22. Secondary IAH or ACS refers to conditions that do not originate from the abdominopelvic region.

- Agree
- Do not agree

Comments (eg to modify definition)

23. Recurrent IAH or ACS refers to the condition in which ACS redevelops following previous surgical or medical treatment of primary or secondary ACS.

- Agree
- Do not agree

Comments (eg to modify definition)

24. For further fine-tuning and classification of IAH/ACS four questions need to be answered. 1. What is the duration of IAH/ACS? 2. Is an intra-abdominal problem responsible for the IAH/ACS? 3. What is the etiology of the IAH/ACS? 4. Is there a local compartment syndrome?

- Agree
- Do not agree

Comments (eg to modify definition)

25. IAH duration can be chronic, acute, subacute or hyperacute

- Agree
- Do not agree

Comments (eg to modify definition)

26. Chronic IAH is defined as IAH that lasts for months or years (eg ovarian tumour, ascites, pregnancy)

- Agree
- Do not agree

Comments (eg to modify definition)

27. acute IAH is defined as IAH that develops within hours (eg ruptured AAA)

- Agree
- Do not agree

Comments (eg to modify definition)

28. subacute IAH is defined as IAH that develops within days (eg fluid overload an capillary leak)

- Agree
- Do not agree

Comments (eg to modify definition)

29. hyperacute IAH is defined as IAH that only lasts for second or minutes (eg coughing, sneezing)

- ◯ Agree
- ◯ Do not agree

Comments (eg to modify definition)

30. APP (abdominal perfusion pressure) = MAP – IAP and should be kept above 60 mmHg.

- ◯ Agree
- ◯ Do not agree

Comments (eg to modify definition)

31. The FG (filtration gradient) is the mechanical force across the glomerulus and equals the difference between the glomerular filtration pressure (GFP) and the proximal tubular pressure (PTP). FG = GFP – PTP, with GFP = MAP - RVP (renal venous pressure).

- ◯ Agree
- ◯ Do not agree

Comments (eg to modify definition)

32. In the presence of IAH, PTP may be assumed to equal RVP and IAP, and thus GFP can be estimated as MAP - IAP. The FG can then be calculated by the formula: FG = MAP - 2*IAP

- ◯ Agree
- ◯ Do not agree

Comments (eg to modify definition)

33. A poly-compartment syndrome is a condition where two or more anatomical compartments have elevated compartmental pressures.

- ◯ Agree
- ◯ Do not agree

Comments (eg to modify definition)

34. There are 4 major body compartments (head, chest, abdomen, and extremities).

- Agree
- Do not agree

Comments (eg to modify definition)

35. Abdominal compliance (Cab) is a measure of the ease of abdominal expansion, which is determined by the elasticity of the abdominal wall and diaphragm. It should be expressed as the change in intra-abdominal volume per change in intra-abdominal pressure.

- Agree
- Do not agree

Comments (eg to modify definition)

36. RAV (respiratory abdominal variation) is an indirect measure of abdominal compliance (Cab) and can be caluclated as IAPei - IAPee (delta IAP)

- Agree
- Do not agree

Comments (eg to modify definition)

37. RAVT (respiratory abdominal variation test) is a nonivasive test assessing RAV during gradual increase in tidal volume (in mechanically ventilated patients) and provides indirect measure of Cab

- Agree
- Do not agree

Comments (eg to modify definition)

38. PAVT (positional abdominal variation test) is a nonivasive test assessing RAV during gradual changes in HOB (also in spontaneous breathing) and provides indirect measure of Cab

- Agree
- Do not agree

Comments (eg to modify definition)

39. APV (abdominal pressure variation) is an indirect measure of Cab and can be calculated as (IAPei - IAPee)/IAPmean

- Agree
- Do not agree

Comments (eg to modify definition)

40. Continuous IAP can be used to keep track of changes in IAP during treatment.

- Agree
- Do not agree

Comments (eg to modify definition)

41. Different techniques exist to perform continuous IAP monitoring (eg gastric, bladder, direct). A gold standard yet needs to be identified.

- Agree
- Do not agree

Comments (eg to modify definition)

42. The open abdomen is one that requires a temporary abdominal closure (TAC) due to the skin and fascia not being closed after laparotomy.

- Agree
- Do not agree

Comments (eg to modify definition)

43. Lateralization of the abdominal wall is the phenomenon where the musculature and fascia of the abdominal wall, most exemplified by the rectus abdominus muscles and their enveloping fascia, move lateraly away from the midine with time.

- Agree
- Do not agree

Comments (eg to modify definition)

44. When left open with a temporary abdominal closure (TAC), the open abdomen should be closed as soon as possible (best within 1 week)

- Agree
- Do not agree

Comments (eg to modify definition)

45. Medical management is defined as a nonsurgical intervention with the purpose to lower increased IAP and consists of 5 treatment options: improvement of Cab, decrease of intra-abdominal volume (IAV), decrease of Intra-luminal Volume, fluid management, organ support.

- Agree
- Do not agree

Comments (eg to modify definition)

46. The four distinct IAH categories are defined as medical, surgical, trauma or burns

- Agree
- Do not agree

Comments (eg to modify definition)

47. Localised IAH and ACS is defined as a local increase in IAP that does not lead to a systemic elevation (eg pelvic trauma, liver or spleen trauma)

- Agree
- Do not agree

Comments (eg to modify definition)

48. Have you heard before from WSACS?

- Yes
- No
- A little
- Say again, what is WSACS?

49. Are you a member of the WSACS?

- Yes
- No

50. Are you aware of the previous WSACS consensus definitions from 2013?

- ◯ Yes
- ◯ No
- ◯ A little (heard from it)

51. Were you involved in previous WSACS consensus definitions development?

- ☐ Yes in 2006
- ☐ Yes in 2013
- ☐ No

52. Are you aware of the WSACS treatment guidelines and recommendations?

- ◯ Yes
- ◯ No
- ◯ A little

53. Have you implemented the WSACS consensus definitions, guidelines and treatment recommendations in clinical practice?

- ◯ Yes
- ◯ No
- ◯ We are working on it

54. What do you think is the future of the Abdominal compartment society (WSACS)?

- ◯ WSACS should continue
- ◯ WSACS can stop now (retire)
- ◯ WSACS should be part of another society
- ◯ I have no clue (undecided)
- ◯ Other

* 55. In what country do you live?

Other (please specify)

56. Would you like to become more actively involved in future WSACS projects?

- ☐ Yes, I would like to become WSACS ambassador
- ☐ Yes, I would like to collaborate for research
- ☐ Yes, I would like to actively participate in future guideline development
- ☐ Other (please specify)
- ☐ Yes, I would like to become WSACS member (free membership)
- ☐ No thanks

57. Please leave your contact details if you want to be more actively involved (mandatory for future feedback - information will be dealt with GDPR proof)

Given and Family Name

Affiliation

City

E-mail address

Table 2. Comparison of agreement among respondents on the basis of their education

	Statements	Physicians, n=737	Nurses, n=254	Trainees, n=40	Others, n=11	p–value
	**Overall**	**89.17(±10.89)**	**88.10(±11.62)**	**88.10(±11.62)**	**89.34(±11.36)**	**0.57**
	**Pathophysiology of IAH**	91.6 (±13.76)	91.5(±12.8)	91.94(±10.34)	91.67(±14.05)	0.99
1	The abdominal cavity/compartment is considered as being primarily fluid in character following Pascal's law.	577(84.2%)	240(94.5%)	43(87.8%)	34(87.2%)	<0.01*
2	Intra–abdominal pressure (IAP) is the steady–state pressure concealed within the abdominal cavity.	594(86.1%)	244(96.4%)	41(83.7%)	35(92.1%)	<0.01*
3	APP = MAP – IAP and should be kept above 60 mmHg.	620 (91.2%)	224(88.2%)	45(93.8%)	34(91.9%)	0.45
4	The FG is the mechanical force across the glomerulus and equals the difference between GFR and PTP. [FG = GFP – PTP, with GFP = MAP– RVP]	643 (95.3%)	229(90.2%)	45(93.8%)	30(85.7%)	<0.01*
5	In the presence of IAH, PTP may be assumed to equal RVP and IAP, and thus GFP can be estimated as MAP–IAP. The FG can then be calculated by the formula: FG = MAP– 2xIAP.	594(89.5%)	226(89%)	41(87.2%)	31(88.6%)	0.97
6	A poly–compartment syndrome is a condition where two or more anatomical compartments have elevated compartmental pressures.	650(95.4%)	236(92.9%)	47(97.9%)	36(97.3%)	0.27
7	There are 4 major body compartments (head, chest, abdomen, and extremities).	642(94.6%)	231(90.9%)	44(91.7%)	35(94.6%)	0.23
8	Abdominal compliance (Cab) is a measure of the ease of abdominal expansion, which is determined by the elasticity of the abdominal wall and diaphragm. It should be expressed as the change in intra–abdominal volume per change in IAP.	659 (96.6%)	236(92.9%)	44(91.7%)	34(94.4%)	0.06
9	Respiratory abdominal variation (RAV) is an indirect measure of Cab and can be calculated as IAPei – IAPee (delta IAP).	610 (91.6%)	223(87.8%)	46 (95.8%)	31(88.6%)	0.18
10	Respiratory abdominal variation test (RAVT) is a non–invasive test assessing RAV during gradual increase in tidal volume (in mechanically ventilated patients) and provides indirect measure of Cab.	614(92.7%)	234(92.1%)	47(97.9%)	29(82.9%)	0.08
11	Positional abdominal variation test (PAVT) is a non–invasive test assessing RAV during gradual changes in HOB (also in spontaneous breathing) and provides indirect measure of Cab.	594(90.3%)	232(91.7%)	44(91.7%)	32(94.1%)	0.81
12	Abdominal pressure variation (APV) is an indirect measure of Cab and can be calculated as (IAPei – IAPee)/IAPmean.	612(92.0%)	231(91.3%)	43 (89.6%)	32(94.1%)	0.88
	**Measurement of intrabdominal pressure**	81.98(±15.32)	83.63(±14.8)	80.61(±15.78)	85.5 (±13.19)	0.21
13	Clinical assessment and estimation of IAP is inaccurate.	505(73.2%)	122(48.0%)	27(56.3%)	21(55.3%)	<0.01*
14	The reference standard for intermittent IAP measurement is via the bladder with a maximal instillation volume of 20–25 mL of sterile saline.	603(87.9%)	224(88.2%)	41(83.7%)	34(87.2%)	0.84
15	The gastric route can be used as alternative for intermittent IAP measurement with a maximal instillation volume of 50–75 mL of water or nutritional fluid.	434(63.6%)	221(87.0%)	40(81.6%)	33 (86.8%)	<0.01*
16	IAP should be expressed in mmHg and measured at end–expiration in the supine position after ensuring that abdominal muscle contractions are absent and with the transducer zeroed at the level of the midaxillary line	645(94.4%)	216(85.0%)	45(91.8%	34 (89.5%)	<0.01*
17	After IAP measurement in the supine position, IAP should also be measured in the "resting" position of the patient, e.g., the normal HOB is at 30–45° position or prone position.	450(65.7%)	223(87.8%)	37(75.5%)	30(83.3%)	<0.01*
18	IAP measurement can also be performed in awake or spontaneously breathing patients.	552(80.5%)	229(90.2%)	40(81.6%)	35(92.1%)	<0.01*

	Statements	Physicians, n=737	Nurses, n=254	Trainees, n=40	Others, n=11	p–value
19	Normal IAP is approximately 5–7 mmHg in critically ill adults.<br>Suggestion to modify as follows: IAP is approximately 5–7 mmHg in healthy adults.	563(81.8%)	218(86.2%)	34(70.8%)	33(89.2%)	0.04*
20	Normal IAP is approximately 10 mmHg in critically ill adults.	529(77.1%)	199(78.7%)	38(79.2%)	27(75.0%)	0.93
21	The normal IAP differs regarding the patient population and anthropometry and can be non–pathologically increased 10–15 mmHg in obese patients, pregnancy, etc.	644(93.6%)	229(90.2%)	42(87.5%)	32(91.4%)	0.18
22	Different techniques exist to perform continuous IAP monitoring (e.g., gastric, bladder, direct). A gold standard yet needs to be identified.	619(90.4%)	227(89.4%)	43(89.6%)	35(92.1%)	0.94
23	Continuous IAP can be used to keep track of changes in IAP during treatment.	635(93.4%)	227(89.4%)	42(87.5%)	36(94.7%)	0.12
	**Definitions**	90.89(±12.86)	88.33(±13.54)	89.58(±15.61)	90.45 (±13.2)	0.07
24	IAH (intra–abdominal hypertension) is defined by a sustained or repeated pathological elevation in IAP > 12 mmHg.	633(92.0%)	224(88.2%)	46(95.8%)	34(91.9%)	0.19
25	Abdominal compartment syndrome (ACS) is an all or nothing phenomenon and defined as a sustained IAP >20 mmHg (with or without an APP < 60 mmHg) that is associated with new organ dysfunction/failure.	622 (90.5%)	215(84.6%)	46(95.8%)	33(89.2%)	0.03*
26	Organ dysfunction/failure is assessed by (a daily) SOFA or equivalent scoring system (qSOFA); organ failure is defined as a SOFA organ system sub–score of >2.	601(87.7%)	221(87.4%)	44(91.7%)	30(85.7%)	0.84
27	Sustained increase in IAP is defined as a pathological value during a minimum of three standardized measurements that are performed 1–2 hours apart for ACS and 4–6 hours apart for IAH.	617(90.1%)	221(87.4%)	44(91.7%	32(88.9%)	0.63
28	IAH is a continuum and graded as follows: Grade I, IAP 12–15 mmHg, Grade II, IAP 16–20 mmHg, Grade III, IAP 21–25 mmHg, and Grade IV, IAP > 25 mmHg.	638(93.3%)	228(89.8%)	43(89.6%)	32(86.5%)	0.16
29	Primary IAH or ACS is a condition associated with injury or disease in the abdominopelvic region that frequently requires early surgical or interventional radiological intervention.	606(88.6%)	213(83.9%)	41(85.4%)	30(85.7%)	0.27
30	Secondary IAH or ACS refers to conditions that do not originate from the abdominopelvic region.	631(92.7%)	216(85.0%)	41(85.4%)	28(80.0%)	<0.01*
31	Recurrent IAH or ACS refers to the condition in which ACS redevelops following previous surgical or medical treatment of primary or secondary ACS.	650(95.0%)	23(92.1%)	45(93.8%)	33(94.3%)	0.40
32	The four distinct IAH categories are defined as medical, surgical, trauma or burns.	626(92.1%)	235(92.5%)	42(87.5%)	35(94.6%)	0.62
33	Localized IAH and ACS is defined as a local increase in IAP that does not lead to a systemic elevation (e.g., pelvic trauma, liver or spleen trauma).	585(86.5%)	230(90.6%)	41(87.2%)	33(91.7%)	0.35
34	IAH duration can be chronic, acute, subacute or hyperacute.	609(89.0%)	226(89.3%)	38(79.2%)	32(88.9%)	0.21
35	Chronic IAH is defined as IAH that lasts for months or years (e.g., ovarian tumor, ascites, pregnancy).	626(91.9%)	229(90.2%)	44(91.7%)	33(94.3%)	0.78
36	Acute IAH is defined as IAH that develops within hours (e.g., ruptured AAA).	661 (96.5%)	220(87.0%)	42(87.5%)	34(94.4%)	<0.01*
37	Subacute IAH is defined as IAH that develops within days (e.g., fluid overload and capillary leak).	655(95.6%)	233(91.7%)	45(93.8%)	34(94.4%)	0.14
38	Hyperacute IAH is defined as IAH that only lasts for second or minutes (e.g., coughing, sneezing).	556 (81.8%)	217(85.4%)	42 (87.5%)	33(89.2%)	0.32
	**Management of IAH/ACS**	94.38(±13.51)	89.06(±17.73)	90 (±15.44)	90.81(±15.99)	<0.01*
39	For further fine–tuning and classification of IAH/ACS four questions need to be answered. 1. What is the duration of IAH/ACS? 2. Is an intra–abdominal problem responsible for the IAH/ACS? 3. What is the etiology of the IAH/ACS? 4. Is there a local compartment syndrome?	642(94.6%)	232(91.7%)	42(87.5%)	33(91.7%)	0.14

	Statements	Physicians, n=737	Nurses, n=254	Trainees, n=40	Others, n=11	p–value
40	The open abdomen is one that requires a TAC due to the skin and fascia not being closed after laparotomy.	645(94.9%)	221(87.0%)	44(91.7%)	31(88.6%)	<0.01*
41	Lateralization of the abdominal wall is the phenomenon where the musculature and fascia of the abdominal wall, most exemplified by the rectus abdominus muscles and their enveloping fascia, move laterally away from the midline with time.	644(95.4%)	231(90.9%)	43(89.6%)	32(91.4%)	0.04
42	When left open with a TAC, the open abdomen should be closed as soon as possible (best within one week).	606(89.9%)	209(82.3%)	43(89.6%)	28(82.4%)	0.01*
43	Medical management is defined as a nonsurgical intervention with the purpose to lower increased IAP and consists of five treatment options: improvement of Cab, decrease of intra–abdominal volume, decrease of intra–luminal volume, fluid management, organ support.	660(96.9%)	237(93.3%)	44(91.7%)	34(97.1%)	0.04*

IAP, intra–abdominal pressure; IAPei, intra–abdominal pressure at end–inspiration; IAPee, intra–abdominal pressure at end–expiration; IAPmean, mean intra–abdominal pressure; APP, abdominal perfusion pressure; MAP, mean arterial pressure; FG, filtration gradient; GFP, glomerular filtration pressure; PTP, proximal tubular pressure; RVP, renal venous pressure; IAH, intraabdominal hypertension; Cab, abdominal compliance; RAV, respiratory abdominal variation; PAVT, positional abdominal variation test; HOB, head of bed; APV, abdominal pressure variation; SD, standard deviation; IAH, intra–abdominal hypertension; ACS, abdominal compartment syndrome ACS; SOFA, sequential organ failure assessment; qSOFA, quick SOFA; TAC, temporary abdominal closure; SD, standard deviation.

Table 3. Comparison of agreement among respondents on the basis of their base specialty

	Statements	Anesthesia, n=369	Internal Medicine, n=131	Surgery, n=138	Pediatrics, n=55	Emergency Medicine, n=44	p–value
	**Overall**	**88.38(±10.12)**	**89.99(±10.95)**	**90.15(±10.35)**	**89.20(±11.73)**	**89.27(±11.83)**	**0.48**
	**Pathophysiology of IAH**	91.11(±12.11)	91.64(±13.67)	93.13(±14.18)	92.41(±12.03)	92.58(±14.89)	0.71
1	The abdominal cavity/compartment is considered as being primarily fluid in character following Pascal's law.	303(82.1%)	111(84.7%)	120(72.7%)	52(94.5%)	38(86.4%)	<0.01*
2	Intra–abdominal pressure (IAP) is the steady–state pressure concealed within the abdominal cavity.	296(80.2%)	120(89.6%)	128(93.4%)	53(96.4%	41(91.1%)	<0.01*
3	APP = MAP – IAP and should be kept above 60 mmHg.	340(93.4%)	118(89.4%)	120(88.9%)	44(83.0%)	41(91.1%)	0.16
4	The FG is the mechanical force across the glomerulus and equals the difference between GFR and PTP. [FG = GFP – PTP, with GFP = MAP– RVP]	349(95.6%)	121(94.5%)	126(95.5%)	51(92.7%)	43(95.6%)	0.04*
5	In the presence of IAH, PTP may be assumed to equal RVP and IAP, and thus GFP can be estimated as MAP–IAP. The FG can then be calculated by the formula: FG = MAP– 2xIAP.	317(88.3%)	112(88.2%)	124(95.4%)	49(89.1%)	41(91.1%)	0.28
6	A poly–compartment syndrome is a condition where two or more anatomical compartments have elevated compartmental pressures.	347(95.3%)	126(96.9%)	132(97.1%)	52(94.5%	42(93.3%)	0.47
7	There are 4 major body compartments (head, chest, abdomen, and extremities).	341(93.7%)	127(96.2%)	128(94.8%)	51(92.7%)	42(93.3%)	0.63
8	Abdominal compliance (Cab) is a measure of the ease of abdominal expansion, which is determined by the elasticity of the abdominal wall and diaphragm. It should be expressed as the change in intra–abdominal volume per change in IAP.	352(96.2%)	126(96.9%)	130(96.3%)	53(96.4%)	42(95.5%)	0.70
9	Respiratory abdominal variation (RAV) is an indirect measure of Cab and can be calculated as IAPei – IAPee (delta IAP).	328(91.6%)	121(93.1%)	118(90.8%	51(92.7%)	41(93.2%)	0.40

	Statements	Anesthesia, n=369	Internal Medicine, n=131	Surgery, n=138	Pediatrics, n=55	Emergency Medicine, n=44	p–value
10	Respiratory abdominal variation test (RAVT) is a non–invasive test assessing RAV during gradual increase in tidal volume (in mechanically ventilated patients) and provides indirect measure of Cab.	341(94.7%)	119(93.0%)	117(91.4%)	50(90.9%)	41(93.2%)	0.43
11	Positional abdominal variation test (PAVT) is a non–invasive test assessing RAV during gradual changes in HOB (also in spontaneous breathing) and provides indirect measure of Cab.	326(90.1%)	109(87.9%)	121(94.5%)	52(94.5%)	41(93.2%)	0.70
12	Abdominal pressure variation (APV) is an indirect measure of Cab and can be calculated as (IAPei – IAPee)/IAPmean.	332(92.2%)	115(89.1%)	120(94.5%)	50(90.9%)	42(93.3%)	0.63
	**Measurement of intrabdominal pressure**	80.37(±15.22)	84.53(±14.84)	82.81(±15.37)	82.61(±16.23)	84.58(±13.79)	0.02*
13	Clinical assessment and estimation of IAP is inaccurate.	241 (65.7%)	105 (78.4%)	109(79.0%)	38(69.1%)	28 (62.2%)	<0.01*
14	The reference standard for intermittent IAP measurement is via the bladder with a maximal instillation volume of 20–25 mL of sterile saline.	318 (86.4%)	122 (91.7%)	118(86.8%)	47(87.0%)	40 (88.9%)	0.74
15	The gastric route can be used as alternative for intermittent IAP measurement with a maximal instillation volume of 50–75 mL of water or nutritional fluid.	227(62.2%)	87(65.9%)	96(70.1%)	39(72.2%)	39(86.7%)	<0.01*
16	IAP should be expressed in mmHg and measured at end–expiration in the supine position after ensuring that abdominal muscle contractions are absent and with the transducer zeroed at the level of the midaxillary line.	343(93.7%)	127(94.8%)	127(95.5%)	49(89.1%)	43(95.6%)	<0.01*
17	After IAP measurement in the supine position, IAP should also be measured in the "resting" position of the patient, e.g., the normal HOB is at 30–45° position or prone position.	238(65.2%)	96(72.2%)	83(61.0%)	40(72.7%)	32(72.7%)	<0.01*
18	IAP measurement can also be performed in awake or spontaneously breathing patients.	301(82.0%)	104(78.2%)	109(79.6%)	48(87.3%)	37(86.0%)	0.07
19	Normal IAP is approximately 5–7 mmHg in critically ill adults.<br>Suggestion to modify as follows: IAP is approximately 5–7 mmHg in healthy adults.	284(77.4%)	116(87.2%)	118(86.1%)	47(85.5%)	34(77.3%)	0.01*
20	Normal IAP is approximately 10 mmHg in critically ill adults.	276(75.0%)	108(82.4%)	109(80.1%)	43(78.2%)	34(75.6%)	0.58
21	The normal IAP differs regarding the patient population and anthropometry and can be non–pathologically increased 10–15 mmHg in obese patients, pregnancy, etc.	342(92.9%)	127(95.5%)	124(91.2%)	52(94.5%)	43(95.6%)	0.40
22	Different techniques exist to perform continuous IAP monitoring (e.g., gastric, bladder, direct). A gold standard yet needs to be identified.	336(91.6%)	118(89.4%)	117(86.7%)	47(85.5%)	41(91.1%)	0.49
23	Continuous IAP can be used to keep track of changes in IAP during treatment.	335(92.0%)	121(93.1%)	127(94.1%)	48(87.3%)	44(97.8%)	0.40
	**Definitions**	90.52(±12.69)	91.28(±13.19)	91.45(±12.74)	90.16(±13.20)	89.72(±12.46)	0.40
24	IAH (intra–abdominal hypertension) is defined by a sustained or repeated pathological elevation in IAP > 12 mmHg.	332(90.5%)	125(94.0%)	127(93.4%)	52(94.5%)	37(82.2%)	0.17
25	Abdominal compartment syndrome (ACS) is an all or nothing phenomenon and defined as a sustained IAP >20 mmHg (with or without an APP < 60 mmHg) that is associated with new organ dysfunction/failure.	337(91.8%)	115(87.8%)	127(92.7%)	47(85.5%)	41(91.1%)	0.09
26	Organ dysfunction/failure is assessed by (a daily) SOFA or equivalent scoring system (qSOFA); organ failure is defined as a SOFA organ system sub–score of >2.	322(88.0%)	112(85.5%)	124(90.5%)	48(88.9%)	38(84.4%)	0.83

Statements		Anesthesia, n=369	Internal Medicine, n=131	Surgery, n=138	Pediatrics, n=55	Emergency Medicine, n=44	p–value
27	Sustained increase in IAP is defined as a pathological value during a minimum of three standardized measurements that are performed 1–2 hours apart for ACS and 4–6 hours apart for IAH.	334(90.8%)	114(89.8%)	126(92.0%)	46(83.6%)	43(95.6%)	0.18
28	IAH is a continuum and graded as follows: Grade I, IAP 12–15 mmHg, Grade II, IAP 16–20 mmHg, Grade III, IAP 21–25 mmHg, and Grade IV, IAP > 25 mmHg.	334(91.3%)	122(93.1%)	132(97.1%)	50(90.9%)	41(93.2%)	0.19
29	Primary IAH or ACS is a condition associated with injury or disease in the abdominopelvic region that frequently requires early surgical or interventional radiological intervention.	332(90.5%)	114(88.4%)	118(86.8%)	47(85.5%)	37(82.2%)	0.17
30	Secondary IAH or ACS refers to conditions that do not originate from the abdominopelvic region.	336(91.8%	115(89.1%)	129(95.6%)	49(89.1%)	39(88.6%)	0.08
31	Recurrent IAH or ACS refers to the condition in which ACS redevelops following previous surgical or medical treatment of primary or secondary ACS.	348(95.1%)	121(92.4%)	130 (96.3%)	51(94.4%)	43(95.6%)	0.53
32	The four distinct IAH categories are defined as medical, surgical, trauma or burns.	331(91.2%)	121(92.4%)	123 (91.1%)	51(92.7%)	43(95.6%)	0.90
33	Localized IAH and ACS is defined as a local increase in IAP that does not lead to a systemic elevation (e.g., pelvic trauma, liver or spleen trauma).	320(88.2%)	117(90.0%)	107 (79.9%)	50(90.9%)	34(77.3%)	<0.01*
34	IAH duration can be chronic, acute, subacute or hyperacute.	316(86.1%)	121(93.8%)	122 (89.7%)	48(87.3%)	39(86.7%)	0.72
35	Chronic IAH is defined as IAH that lasts for months or years (e.g., ovarian tumor, ascites, pregnancy).	331(90.7%)	122(93.8%)	128 (94.1%)	51(92.7%)	41(91.1%)	<0.01*
36	Acute IAH is defined as IAH that develops within hours (e.g., ruptured AAA).	346(94.3%)	125(96.2%)	133 (98.5%)	53(96.4%)	43(95.6%)	0.33
37	Subacute IAH is defined as IAH that develops within days (e.g., fluid overload and capillary leak).	351(95.6%)	124(94.7%)	130 (95.6%)	50(90.9%)	44(97.8%)	0.34
38	Hyperacute IAH is defined as IAH that only lasts for second or minutes (e.g., coughing, sneezing).	302(82.5%)	114(87.0%)	105(78.9%)	49(89.1%)	40(88.9%)	0.41
**Management of IAH/ACS**		93.34(±14.10)	94.70(±13.5)	95.07(±12.05)	93.09(±15.02)	91.11(±18.37)	<0.01*
39	For further fine–tuning and classification of IAH/ACS four questions need to be answered. 1. What is the duration of IAH/ACS? 2. Is an intra–abdominal problem responsible for the IAH/ACS? 3. What is the etiology of the IAH/ACS? 4. Is there a local compartment syndrome?	336(92.6%)	126(97.7%)	124(91.9%)	51(92.7%)	42(93.3%)	0.44
40	The open abdomen is one that requires a TAC due to the skin and fascia not being closed after laparotomy.	345(94.3%)	122(95.4%)	130(95.6%)	52(94.5%)	39(88.6%)	<0.01*
41	Lateralization of the abdominal wall is the phenomenon where the musculature and fascia of the abdominal wall, most exemplified by the rectus abdominus muscles and their enveloping fascia, move laterally away from the midline with time.	341(93.7%)	120(93.8%)	132(98.5%)	50(90.9%)	41(93.2%)	0.23
42	When left open with a TAC, the open abdomen should be closed as soon as possible (best within one week).	328(90.1%)	116(89.2%)	124(91.9%)	51(92.7%)	36(81.8%)	0.01*
43	Medical management is defined as a nonsurgical intervention with the purpose to lower increased IAP and consists of five treatment options: improvement of Cab, decrease of intra–abdominal volume, decrease of intra–luminal volume, fluid management, organ support.	351(95.9%)	126(96.9%)	133(97.8%)	52(94.5%)	43(97.7%)	0.53

IAP, intra–abdominal pressure; IAPei, intra–abdominal pressure at end–inspiration; IAPee, intra–abdominal pressure at end–expiration; IAPmean, mean intra–abdominal pressure; APP, abdominal perfusion pressure; MAP, mean arterial pressure; FG, filtration gradient; GFP, glomerular filtration pressure; PTP, proximal tubular pressure; RVP, renal venous pressure; IAH, intraabdominal hypertension; Cab, abdominal compliance; RAV, respiratory abdominal variation; PAVT, positional abdominal variation test; HOB, head of bed; APV, abdominal pressure variation; SD, standard deviation; IAH, intra–abdominal hypertension; ACS, abdominal compartment syndrome ACS; SOFA, sequential organ failure assessment; qSOFA, quick SOFA; TAC, temporary abdominal closure; SD, standard deviation.

Table 4. Comparison of agreement among respondents on the basis of their duration of work experience

Statements		0–5 years n=391	6–10 years n=186	11–15 years n=137	>15 years n=308	p–value
**Overall**		**88.35(±11.09)**	**88.25(±11.23)**	**90.17(±10.26)**	**88.90(±11.02)**	**0.11**
**Pathophysiology of IAH**		90.99(±12.7)	91.48(±13.48)	90.74(±15.03)	92.90(±13.02)	0.22
1	The abdominal cavity/compartment is considered as being primarily fluid in character following Pascal's law.	348 (89.0%)	164 (88.2%)	116 (84.7%)	261 (84.7%)	0.30
2	Intra–abdominal pressure (IAP) is the steady–state pressure concealed within the abdominal cavity.	342 (87.7%)	164 (88.6%)	127 (92.0%	277 (89.1%)	0.59
3	APP = MAP – IAP and should be kept above 60 mmHg.	350 (90.2%)	159 (88.3%)	122 (91.0%)	285 (91.9%)	0.61
4	The FG is the mechanical force across the glomerulus and equals the difference between GFR and PTP. [FG = GFP – PTP, with GFP = MAP– RVP]	356 (92.5%)	167 (93.3%)	124 (92.5%)	293 (95.4%)	0.42
5	In the presence of IAH, PTP may be assumed to equal RVP and IAP, and thus GFP can be estimated as MAP–IAP. The FG can then be calculated by the formula: FG = MAP– 2xIAP.	336 (87.7%)	164 (91.6%)	117 (89.3%)	269 (89.7%)	0.57
6	A poly–compartment syndrome is a condition where two or more anatomical compartments have elevated compartmental pressures.	362 (93.8%)	174 (95.6%)	131 (95.6%)	296 (96.1%)	0.52
7	There are 4 major body compartments (head, chest, abdomen, and extremities).	358 (92.5%)	165 (90.7%)	131 (95.6%)	296 (95.8%)	0.08
8	Abdominal compliance (Cab) is a measure of the ease of abdominal expansion, which is determined by the elasticity of the abdominal wall and diaphragm. It should be expressed as the change in intra–abdominal volume per change in IAP.	363 (94.0%)	177 (97.3%)	130 (94.2%)	297 (96.7%)	0.19
9	Respiratory abdominal variation (RAV) is an indirect measure of Cab and can be calculated as IAPei – IAPee (delta IAP).	343 (88.9%)	159 (87.8%)	123 (92.5%)	279 (94.3%)	0.04*
10	Respiratory abdominal variation test (RAVT) is a non–invasive test assessing RAV during gradual increase in tidal volume (in mechanically ventilated patients) and provides indirect measure of Cab.	358 (93.0%)	169 (93.4%)	119 (89.5%)	276 (92.9%)	0.54
11	Positional abdominal variation test (PAVT) is a non–invasive test assessing RAV during gradual changes in HOB (also in spontaneous breathing) and provides indirect measure of Cab.	351 (91.6%)	162 (90.5%)	110 (84.0%)	277 (93.3%)	0.02*
12	Abdominal pressure variation (APV) is an indirect measure of Cab and can be calculated as (IAPei – IAPee)/IAPmean.	349 (90.9%)	164 (91.1%)	117 (88.6%)	282 (94.6%)	0.14
**Measurement of intrabdominal pressure**		82.63(±15.06)	81.45(±15.1)	81.42(±15.45)	83.46(±15.04)	0.41
13	Clinical assessment and estimation of IAP is inaccurate.	218 (56.0%)	108 (58.1%)	96 (69.6%)	249 (80.1%)	<0.01*
14	The reference standard for intermittent IAP measurement is via the bladder with a maximal instillation volume of 20–25 mL of sterile saline.	337 (86.2%)	165 (89.7%)	124 (89.9%)	271 (87.7%)	0.55
15	The gastric route can be used as alternative for intermittent IAP measurement with a maximal instillation volume of 50–75 mL of water or nutritional fluid.	301 (77.6%)	136 (74.3%)	88 (64.7%)	200(64.7%)	<0.01*

	Statements	0–5 years n=391	6–10 years n=186	11–15 years n=137	>15 years n=308	p–value
16	IAP should be expressed in mmHg and measured at end–expiration in the supine position after ensuring that abdominal muscle contractions are absent and with the transducer zeroed at the level of the midaxillary line.	348 (90.2%)	164 (89.1%)	126 (91.3%)	297(95.8%)	<0.01*
17	After IAP measurement in the supine position, IAP should also be measured in the "resting" position of the patient, e.g., the normal HOB is at 30–45° position or prone position.	304 (78.4%)	132 (71.7%)	92 (67.2%)	208(67.5%)	<0.01*
18	IAP measurement can also be performed in awake or spontaneously breathing patients.	332 (85.3%)	151 (81.6%)	105 (77.2%)	261(84.2%)	0.15
19	Normal IAP is approximately 5–7 mmHg in critically ill adults.<br>Suggestion to modify as follows: IAP is approximately 5–7 mmHg in healthy adults.	318 (82.0%)	153 (83.2%)	118 (85.5%)	255 (82.5%)	0.82
20	Normal IAP is approximately 10 mmHg in critically ill adults.	308 (79.4%)	138 (75.4%)	103 (75.2%)	238 (77.3%)	0.64
21	The normal IAP differs regarding the patient population and anthropometry and can be non–pathologically increased 10–15 mmHg in obese patients, pregnancy, etc.	355 (91.5%)	169 (91.4%)	127 (92.7%)	290 (94.2%)	0.55
22	Different techniques exist to perform continuous IAP monitoring (e.g., gastric, bladder, direct). A gold standard yet needs to be identified.	351 (90.5%)	166 (90.7%)	124 (89.9%)	277 (89.4%)	0.95
23	Continuous IAP can be used to keep track of changes in IAP during treatment.	352 (91.4%)	164 (90.1%)	127 (92.0%)	292 (94.5%)	0.30
	**Definitions**	89.53(±13.34)	89.48(±13.14)	90.31(±15.17)	91.49(±11.88)	0.21
24	IAH (intra–abdominal hypertension) is defined by a sustained or repeated pathological elevation in IAP > 12 mmHg.	347 (89.4%)	169 (91.8%)	129 (93.5%)	286 (92.3%)	0.40
25	Abdominal compartment syndrome (ACS) is an all or nothing phenomenon and defined as a sustained IAP >20 mmHg (with or without an APP < 60 mmHg) that is associated with new organ dysfunction/failure.	348 (89.7%)	160 (87.9%)	121 (87.7%)	282 (90.7%)	0.70
26	Organ dysfunction/failure is assessed by (a daily) SOFA or equivalent scoring system (qSOFA); organ failure is defined as a SOFA organ system sub–score of >2.	341 (88.1%)	156 (85.7%)	123 (89.8%)	271 (88.0%)	0.73
27	Sustained increase in IAP is defined as a pathological value during a minimum of three standardized measurements that are performed 1–2 hours apart for ACS and 4–6 hours apart for IAH.	343 (88.9%)	165 (90.7%)	118 (86.1%)	282 (91.0%)	0.42
28	IAH is a continuum and graded as follows: Grade I, IAP 12–15 mmHg, Grade II, IAP 16–20 mmHg, Grade III, IAP 21–25 mmHg, and Grade IV, IAP > 25 mmHg.	347 (89.2%)	167 (91.3%)	127 (93.4%)	293 (95.1%)	0.03*
29	Primary IAH or ACS is a condition associated with injury or disease in the abdominopelvic region that frequently requires early surgical or interventional radiological intervention.	335 (86.8%)	151 (82.5%)	125 (91.2%)	273 (88.6%)	0.10
30	Secondary IAH or ACS refers to conditions that do not originate from the abdominopelvic region.	330 (85.7%)	167 (91.3%)	126 (92.0%)	288 (94.1%)	<0.01*
31	Recurrent IAH or ACS refers to the condition in which ACS redevelops following previous surgical or medical treatment of primary or secondary ACS.	358 (92.7%)	168 (92.3%)	131 (95.6%)	297 (96.4%)	0.11
32	The four distinct IAH categories are defined as medical, surgical, trauma or burns.	352 (90.7%)	166 (92.7%)	127 (92.7%)	287 (93.2%)	0.64
33	Localized IAH and ACS is defined as a local increase in IAP that does not lead to a systemic elevation (e.g., pelvic trauma, liver or spleen trauma).	340 (88.1%)	160 (89.4%)	117 (85.4%)	266 (87.2%)	0.74
34	IAH duration can be chronic, acute, subacute or hyperacute.	343 (88.6%)	163 (88.6%)	119 (87.5%)	274 (89.3%)	0.96
35	Chronic IAH is defined as IAH that lasts for months or years (e.g., ovarian tumor, ascites, pregnancy).	349 (90.9%)	168 (91.3%)	123 (90.4%)	286 (93.2%)	0.69
36	Acute IAH is defined as IAH that develops within hours (e.g., ruptured AAA).	356 (92.2%)	166 (90.2%)	126 (92.0%)	303 (98.4%)	<0.01*
37	Subacute IAH is defined as IAH that develops within days (e.g., fluid overload and capillary leak).	361 (93.3%)	173 (94.0%)	128 (93.4%)	298 (96.8%)	0.21

	Statements	0–5 years n=391	6–10 years n=186	11–15 years n=137	>15 years n=308	p–value
38	Hyperacute IAH is defined as IAH that only lasts for second or minutes (e.g., coughing, sneezing).	341 (88.1%)	148 (80.9%)	114 (84.4%)	241 (78.5%)	<0.01*
**Management of IAH/ACS**		90.76(±17.1)	92.28(±14.90)	93.33(±14.96)	95.18(±11.31)	<0.01*
39	For further fine–tuning and classification of IAH/ACS four questions need to be answered. 1. What is the duration of IAH/ACS? 2. Is an intra–abdominal problem responsible for the IAH/ACS? 3. What is the etiology of the IAH/ACS? 4. Is there a local compartment syndrome?	350 (90.7%)	171 (93.4%)	131 (96.3%)	290 (95.4%)	0.04*
40	The open abdomen is one that requires a TAC due to the skin and fascia not being closed after laparotomy.	349 (90.4%)	167 (92.3%)	129 (93.5%)	291 (95.1%)	0.13
41	Lateralization of the abdominal wall is the phenomenon where the musculature and fascia of the abdominal wall, most exemplified by the rectus abdominus muscles and their enveloping fascia, move laterally away from the midline with time.	353 (91.9%)	167 (93.8%)	128 (93.4%)	295 (96.4%)	0.11
42	When left open with a TAC, the open abdomen should be closed as soon as possible (best within one week).	336 (86.8%)	156 (86.2%)	120 (87.6%)	271 (89.7%)	0.61
43	Medical management is defined as a nonsurgical intervention with the purpose to lower increased IAP and consists of five treatment options: improvement of Cab, decrease of intra–abdominal volume, decrease of intra–luminal volume, fluid management, organ support.	363 (93.8%)	171 (95.0%)	132 (95.7%)	304 (99.0%)	<0.01*

IAP, intra–abdominal pressure; IAPei, intra–abdominal pressure at end–inspiration; IAPee, intra–abdominal pressure at end–expiration; IAPmean, mean intra–abdominal pressure; APP, abdominal perfusion pressure; MAP, mean arterial pressure; FG, filtration gradient; GFP, glomerular filtration pressure; PTP, proximal tubular pressure; RVP, renal venous pressure; IAH, intraabdominal hypertension; Cab, abdominal compliance; RAV, respiratory abdominal variation; PAVT, positional abdominal variation test; HOB, head of bed; APV, abdominal pressure variation; SD, standard deviation; IAH, intra–abdominal hypertension; ACS, abdominal compartment syndrome ACS; SOFA, sequential organ failure assessment; qSOFA, quick SOFA; TAC, temporary abdominal closure; SD, standard deviation.

Table 5. Comparison of agreement among respondents on the basis of their work profile and collaboration in the previous WSACS guidelines.

	Statements	Intensivist, n=486	Non-intensivist, n=531	p value	Collaborators of previous guidelines, n=111	Non-collaborators n=931	p–value
**Overall mean (±SD) agreement**		**89.1(±10.4)**	**88.68(±11.6)**	**0.55**	**91.04(±9.53)**	**88.66(±11.13)**	**0.03***
**Domain: Pathophysiology of IAH, mean (±SD) agreement**		91.37(±13.47)	91.85(±13.02)	0.56	93.82(±10.07)	91.43(±13.49)	0.07
1	The abdominal cavity/compartment is considered as being primarily fluid in character following Pascal's law.	401(82.3%)	484 (91.1%)	<0.01*	98 (89.1%)	791 (86.8%)	0.65
2	Intra–abdominal pressure (IAP) is the steady–state pressure concealed within the abdominal cavity.	421 (85.6%)	485 (91.9%)	<0.01*	105 (94.6%)	804 (88.2%	0.04*
3	APP = MAP – IAP and should be kept above 60 mmHg.	436 (90.1%)	477 (90.9%)	0.67	103 (95.4%)	816 (90.0%)	0.08
4	The FG is the mechanical force across the glomerulus and equals the difference between GFR and PTP. [FG = GFP – PTP, with GFP = MAP– RVP]	454 (94.2%)	482 (92.9%)	0.40	105 (96.3%)	840 (93.4%)	0.30

Statements		Intensivist, n=486	Non-intensivist, n=531	p value	Collaborators of previous guidelines, n=111	Non-collaborators n=931	p–value
5	In the presence of IAH, PTP may be assumed to equal RVP and IAP, and thus GFP can be estimated as MAP–IAP. The FG can then be calculated by the formula: FG = MAP–2xIAP.	454 (96.4%)	482 (93.1%)	0.47	99 (90.0%)	791 (89.3%)	0.28
6	A poly–compartment syndrome is a condition where two or more anatomical compartments have elevated compartmental pressures.	468 (96.5%)	492 (93.9%)	0.05	103 (93.6%)	864 (95.4%)	0.42
7	There are 4 major body compartments (head, chest, abdomen, and extremities).	460 (94.7%)	486 (92.6%)	0.18	100 (91.7%)	849 (93.8%)	0.41
8	Abdominal compliance (Cab) is a measure of the ease of abdominal expansion, which is determined by the elasticity of the abdominal wall and diaphragm. It should be expressed as the change in intra–abdominal volume per change in IAP.	472 (97.1%)	491 (93.9%)	0.01*	107 (97.3%)	864 (95.4%)	0.36
9	Respiratory abdominal variation (RAV) is an indirect measure of Cab and can be calculated as IAPei – IAPee (delta IAP).	444 (93.9%)	456 (87.9%)	<0.01*	103 (94.5%)	805 (90.4%)	0.17
10	Respiratory abdominal variation test (RAVT) is a non–invasive test assessing RAV during gradual increase in tidal volume (in mechanically ventilated patients) and provides indirect measure of Cab.	441 (92.8%)	477 (92.3%)	0.73	104 (96.3%)	818 (92.2%)	0.13
11	Positional abdominal variation test (PAVT) is a non–invasive test assessing RAV during gradual changes in HOB (also in spontaneous breathing) and provides indirect measure of Cab.	422 (89.2%)	476 (92.4%)	0.10	100 (92.6%)	800 (90.8%)	0.54
12	Abdominal pressure variation (APV) is an indirect measure of Cab and can be calculated as (IAPei – IAPee)/IAPmean.	439 (92.2%)	470 (91.4%)	0.65	102 (93.6%)	813 (91.6%)	0.58
**Domain: Measurement of intrabdominal pressure, mean (±SD) agreement**		81.64(±.97)	83.24(±15.28)	0.09	86.26(±14.19)	82.01(±15.18)	<0.01*
13	Clinical assessment and estimation of IAP is inaccurate.	370(75.4%)	298(56.3%)	<0.01*	75 (67.6%)	596 (65.4%)	0.64
14	The reference standard for intermittent IAP measurement is via the bladder with a maximal instillation volume of 20–25 mL of sterile saline.	441(90.4%)	452(85.3%)	0.01*	101 (92.7%)	795 (87.1%)	0.12
15	The gastric route can be used as alternative for intermittent IAP measurement with a maximal instillation volume of 50–75 mL of water or nutritional fluid.	288(59.5%)	435(82.2%)	<0.01*	91 (82.7%)	633 (69.8%)	<0.01*
16	IAP should be expressed in mmHg and measured at end–expiration in the supine position after ensuring that abdominal muscle contractions are absent and with the transducer zeroed at the level of the midaxillary line	463 (95.1%)	468 (88.8%)	<0.01*	102 (92.7%)	834 (91.7%)	0.85
17	After IAP measurement in the supine position, IAP should also be measured in the "resting" position of the patient, e.g., the normal HOB is at 30–45° position or prone position.	309 (63.8%)	423 (80.0%)	<0.01*	87 (79.1%)	649 (71.5%)	0.11
18	IAP measurement can also be performed in awake or spontaneously breathing patients.	389 (79.9%)	457 (86.4%)	<0.01*	101 (91.8%)	750 (82.3%	0.01*
19	Normal IAP is approximately 5–7 mmHg in critically ill adults.<br>Suggestion to modify as follows: IAP is approximately 5–7 mmHg in healthy adults.	391 (80.1%)	449 (85.2%)	0.03*	93 (84.5%)	752 (82.6%)	0.69
20	Normal IAP is approximately 10 mmHg in critically ill adults.	376 (77.0%)	409 (77.9%)	0.75	91 (82.0%)	698 (77.0%)	0.28
21	The normal IAP differs regarding the patient population and anthropometry and can be non–pathologically increased 10–15 mmHg in obese patients, pregnancy, etc.	458 (93.7%	479 (91.2%)	0.15	99 (90.8%)	823 (90.1%)	0.82

Statements		Intensivist, n=486	Non-intensivist, n=531	p value	Collaborators of previous guidelines, n=111	Non-collaborators n=931	p–value
22	Different techniques exist to perform continuous IAP monitoring (e.g., gastric, bladder, direct). A gold standard yet needs to be identified.	438 (89.4%)	476 (90.7%)	0.53	101 (91.0%	841 (92.5%)	0.57
23	Continuous IAP can be used to keep track of changes in IAP during treatment.	450 (93.0%)	481 (91.4%)	0.37	103 (93.6%)	834 (92.0%)	0.54
**Domain: Definitions, mean (±SD) agreement**		90.98(±12.44)	89.51(±13.78)	0.07	91.72(±11.96)	90.06(±13.25)	0.21
24	IAH (intra–abdominal hypertension) is defined by a sustained or repeated pathological elevation in IAP > 12 mmHg.	452 (92.1%)	475 (90.5%)	0.37	101 (91.0%)	833 (91.4%)	0.87
25	Abdominal compartment syndrome (ACS) is an all or nothing phenomenon and defined as a sustained IAP >20 mmHg (with or without an APP < 60 mmHg) that is associated with new organ dysfunction/failure.	433 (88.4%)	474 (90.3%)	0.32	97 (88.2%)	818 (89.7%)	0.63
26	Organ dysfunction/failure is assessed by (a daily) SOFA or equivalent scoring system (qSOFA); organ failure is defined as a SOFA organ system sub–score of >2.	420 (86.2%)	466 (89.1%)	0.17	101 (91.0%)	792 (87.4%)	0.28
27	Sustained increase in IAP is defined as a pathological value during a minimum of three standardized measurements that are performed 1–2 hours apart for ACS and 4–6 hours apart for IAH.	440 (90.2%)	464 (88.7%)	0.45	101 (91.8%)	809 (89.2%)	0.40
28	IAH is a continuum and graded as follows: Grade I, IAP 12–15 mmHg, Grade II, IAP 16–20 mmHg, Grade III, IAP 21–25 mmHg, and Grade IV, IAP > 25 mmHg.	456 (93.8%)	474 (90.1%)	0.04*	103 (92.8%)	835 (92.0%)	0.76
29	Primary IAH or ACS is a condition associated with injury or disease in the abdominopelvic region that frequently requires early surgical or interventional radiological intervention.	430 (88.5%)	449 (85.7%)	0.19	98 (88.3%)	790 (87.2%)	0.74
30	Secondary IAH or ACS refers to conditions that do not originate from the abdominopelvic region.	448 (92.6%)	460 (88.0%)	0.01*	97 (88.2%)	816 (90.3%)	0.49
31	Recurrent IAH or ACS refers to the condition in which ACS redevelops following previous surgical or medical treatment of primary or secondary ACS.	469 (96.3%)	481 (92.1%)	<0.01*	107 (97.3%)	850 (93.8%)	0.14
32	The four distinct IAH categories are defined as medical, surgical, trauma or burns.	445 (92.1%)	483 (92.0%)	0.94	104 (94.5%)	831 (91.7%)	0.30
33	Localized IAH and ACS is defined as a local increase in IAP that does not lead to a systemic elevation (e.g., pelvic trauma, liver or spleen trauma).	420 (87.1%)	461 (88.3%)	0.57	97 (89.0%)	790 (87.6%)	0.67
34	IAH duration can be chronic, acute, subacute or hyperacute.	437 (89.9%)	459 (87.6%)	0.24	101 (91.8%)	802 (88.4%)	0.29
35	Chronic IAH is defined as IAH that lasts for months or years (e.g., ovarian tumor, ascites, pregnancy).	451 (93.2%)	473 (90.4%)	0.11	105 (95.5%)	824 (91.2%)	0.12
36	Acute IAH is defined as IAH that develops within hours (e.g., ruptured AAA).	468 (96.1%)	479 (91.4%)	<0.01*	102 (93.6%)	852 (93.7%	0.95
37	Subacute IAH is defined as IAH that develops within days (e.g., fluid overload and capillary leak).	470 (96.5%)	486 (92.6%)	<0.01*	104 (94.5%)	860 (94.6%)	0.98
38	Hyperacute IAH is defined as IAH that only lasts for second or minutes (e.g., coughing, sneezing).	397 (82.4%)	445 (84.6%)	0.34	97 (88.2%)	749 (82.7%)	0.14
**Domain: Management. mean (±SD) agreement**		94.69(±11.93)	90.84(±17.12)	<0.01*	92.91(±16.16)	92.72(±14.75)	0.90

Statements		Intensivist, n=486	Non-intensivist, n=531	p value	Collaborators of previous guidelines, n=111	Non-collaborators n=931	p–value
39	For further fine–tuning and classification of IAH/ACS four questions need to be answered. 1. What is the duration of IAH/ACS? 2. Is an intra–abdominal problem responsible for the IAH/ACS? 3. What is the etiology of the IAH/ACS? 4. Is there a local compartment syndrome?	461 (95.6%)	477 (91.2%)	<0.01*	104 (95.4%)	842 (93.2%)	0.39
40	The open abdomen is one that requires a TAC due to the skin and fascia not being closed after laparotomy.	465 (95.9%)	468 (89.5%)	<0.01*	101 (92.7%)	837 (92.5%)	0.95
41	Lateralization of the abdominal wall is the phenomenon where the musculature and fascia of the abdominal wall, most exemplified by the rectus abdominus muscles and their enveloping fascia, move laterally away from the midline with time.	455 (94.6%)	486 (93.1%)	0.33	103 (95.4%)	845 (93.8%)	0.51
42	When left open with a TAC, the open abdomen should be closed as soon as possible (best within one week).	430 (89.6%)	449 (85.9%)	0.07	94 (86.2%)	789 (87.9%)	0.63
43	Medical management is defined as a nonsurgical intervention with the purpose to lower increased IAP and consists of five treatment options: improvement of Cab, decrease of intra–abdominal volume, decrease of intra–luminal volume, fluid management, organ support.	473 (97.5%)	493 (94.3%)	0.01*	104 (94.5%)	869 (95.9%)	0.45

IAP, intra–abdominal pressure; IAPei, intra–abdominal pressure at end–inspiration; IAPee, intra–abdominal pressure at end–expiration; IAPmean, mean intra–abdominal pressure; APP, abdominal perfusion pressure; MAP, mean arterial pressure; FG, filtration gradient; GFP, glomerular filtration pressure; PTP, proximal tubular pressure; RVP, renal venous pressure; IAH, intraabdominal hypertension; Cab, abdominal compliance; RAV, respiratory abdominal variation; PAVT, positional abdominal variation test; HOB, head of bed; APV, abdominal pressure variation; SD, standard deviation; IAH, intra–abdominal hypertension; ACS, abdominal compartment syndrome ACS; SOFA, sequential organ failure assessment; qSOFA, quick SOFA; TAC, temporary abdominal closure; SD, standard deviation; WSACS: abdominal compartment society.

Appendix Commentaries: Relevant comments from the respondents on statements

Statement 1: *The abdominal cavity/compartment is considered as being primarily fluid in character following Pascal's law* (overall agreement 86.7%, 33 comments).

The respondents expressed the necessity of incorporating different tissue densities, compliances, and water contents into this definition, indicating a more complex composition than solely fluid–based. They highlighted that the abdominal cavity comprises various elements, such as gas, fluid, stool, and abnormal masses, challenging the oversimplified notion of primarily fluid character. Additionally, the respondents noted evidence suggesting that the IAP is not uniform across every part of the abdomen, further suggesting uniform fluidity. Some researchers have suggested that Pascal's law, which primarily concerns enclosed fluids in incompressible or highly compressible states, may not directly apply to the abdominal cavity, which contains solid or semisolid structures that behave differently under pressure. Furthermore, respondents mentioned the variability of abdominal behavior among individuals, the influence of tissue compliance, and the impact of interventions such as ventilation. These observations collectively underscore the complexity and heterogeneity of the composition and dynamics of the abdominal cavity, challenging the simplistic application of fluid mechanics principles such as Pascal's law in this anatomical context.

Statement 2: *Intra–abdominal pressure (IAP) is the steady–state pressure concealed within the abdominal cavity* (overall agreement 88.3%, 29 comments).

The respondents noted that the pressure fluctuates and has a waveform associated with respiration or ventilation and cardiac output, challenging the notion that it is strictly steady–state. They emphasized the importance of recording the pressure in a relaxed patient at the end–expiration to obtain a more accurate representation. Additionally, comments highlighted that IAP reflects a "one–time" number rather than a continuous steady–state pressure, particularly in patients who are not on neuromuscular blockade. In such patients, there are identifiable peak, mean, and minimum pressures, indicating variability rather than steadiness. Furthermore, respondents noted that the IAP as a variable depends on factors such as the nature of the abdominal content or any pathological state present, further contesting the idea of steady–state pressure. Some mentioned that physiological and pathological conditions can modify the IAP, with variations based on hydration, elimination, and the presence of adhesions or scars. These observations collectively underscore the dynamic and multifactorial nature of IAP, challenging the notion that it is solely steady–state within the abdominal cavity.

Statement 3: *Abdominal perfusion pressure (APP) is the difference between MAP and IAP and should be kept above 60 mmHg* (overall agreement, 89.8%; 56 comments).

The respondents expressed uncertainty and scepticism regarding the validity and utility of this parameter in clinical practice. They highlighted the lack of continuous monitoring of the IAP, making it challenging to consistently maintain the APP above 60 mmHg, especially in critically ill patients. Additionally, the comments suggested that lower targets might be acceptable in specific patient

populations or clinical scenarios. The respondents emphasized the need for individualized approaches and considered factors such as age, organ function assessment, and the underlying pathophysiology. Some respondents questioned the evidence supporting the recommendation to keep the APP above 60 mmHg, particularly in the absence of precise data on its impact on organ perfusion and patient outcomes. Concerns have been raised about the potential for overtreatment or unintended consequences associated with strict adherence to this target, especially without strong empirical evidence. Furthermore, respondents noted the variability in clinical conditions and the lack of generalizable information supporting the arbitrary value of 60 mmHg. The recommendation from the 2013 WSACS guidelines not to use this parameter probably further contributed to doubt among respondents, who called for clearer guidance and evidence supporting its clinical utility. These observations collectively underscore the complexity and uncertainty surrounding the use of APP as a therapeutic target in clinical practice, highlighting the need for further research and clarification in this area.

Statement 4: *The filtration gradient (FG) is the mechanical force across the glomerulus and equals the difference between glomerulus filtration pressure (GFP) and proximal tubular pressure (PTP), further GFP is the difference between mean arterial pressure (MAP) and renal venous pressure (RVP)* (overall agreement 92.9%, 31 comments).

From the comments on this statement, the sentiment leans toward scepticism and concerns about practicality. The respondents expressed doubts about the relevance and utility of the formula in clinical practice, with several suggesting that it might need to be

more practical for bedside clinicians. Concerns were raised about obtaining these numbers and the need for more clarity on how each component would be measured in a hospital setting. Some respondents emphasized the complexity of renal autoregulation, especially in critically ill conditions, making the application of such a simple formula challenging. Additionally, there were comments questioning the accuracy and applicability of the formula, particularly regarding the calculation of GFP as MAP–RVP. The respondents highlighted discrepancies and potential errors in the formula, suggesting that the GFP does not equal the MAP–RVP, as stated. Moreover, the practicality of measuring these parameters at the bedside was questioned, leading some respondents to suggest deleting the formula altogether. Overall, the comments indicate a need for further clarification, education, and empirical evidence supporting the use of this formula in clinical practice.

Statement 5: *In the presence of IAH, PTP may be assumed to equal RVP and IAP, and thus GFP can be estimated as difference between MAP and IAP. The FG can then be calculated by the formula, MAP– 2*IAP* (overall agreement 87.9%, 45 comments).

There was a notable trend of uncertainty about the practicality and usefulness of the proposed formula in clinical practice. Several comments express concerns about the complexity of the derivation and the need for more clarity regarding the variables involved, such as PTP and GFP. The respondents questioned the clinical application of these calculations and emphasized the need for validation and verification of the proposed formula. Some suggest that the formula may be unnecessary and could add confusion rather than clarity to clinical decision-making. Overall, there is a consensus among the comments that further research and evidence

are needed to support the validity and utility of the formula in clinical practice. Many respondents expressed a need for simpler concepts and clearer clinical applications before considering the adoption of such calculations at the bedside.

Statement 6: *A poly–compartment syndrome is a condition where two or more anatomical compartments have elevated compartmental pressures* (overall agreement 94.5%, 16 comments).

The respondents acknowledged the potential utility of the concept of poly-compartment syndrome, while some proposed its inclusion in a grading system for ACS, others expressed uncertainty about its practical application or suggested the use of alternative terminology such as "multicompartment." Additionally, there were comments questioning the relevance or usefulness of the concept, indicating a need for further clarification or discussion to address any concerns or uncertainties surrounding its implementation.

Statement 7: *There are 4 major body compartments (head, chest, abdomen, and extremities)* (overall agreement 93%, 26 comments).

Some respondents suggested considering additional compartments, such as the retroperitoneum, pelvis, ocular, pericardial, and intravascular spaces, indicating potential oversimplification and clinical irrelevance. There were also suggestions to differentiate between the retroperitoneal and peritoneal spaces within the abdomen and to acknowledge the presence of multiple compartments within the extremities. These comments highlight the complexity and diversity of anatomical compartments that warrant consideration beyond the four major compartments initially proposed.

Statement 8: *Abdominal compliance (Cab) is a measure of the ease of abdominal expansion, which is determined by the elasticity of the abdominal wall and diaphragm. It should be expressed as the change in intra–abdominal volume per change in IAP* (overall agreement 95%, 22 comments).

The concept of Cab received an overall agreement of 95% among respondents. However, several respondents expressed concerns regarding the practicality of measuring intra–abdominal volume and determining Cab at the bedside, questioning its clinical utility. Some respondents sought clarification on how intra–abdominal volume is measured and how Cab can be practically calculated. Others have raised issues about the lack of validated clinical monitoring tools for assessing abdominal compliance and have suggested considering individual factors such as abdominal wall thickness and composition. These comments underscore the need for further clarification and the development of practical methods for assessing abdominal compliance in clinical settings.

Statement 9: *Respiratory abdominal variation (RAV) is an indirect measure of Cab and can be calculated as the difference between intra-abdominal pressure at end-inspiration and end-expiration, IAPei – IAPee (delta IAP)* (overall agreement 89.4%, 41 comments).

Few respondents expressed uncertainty regarding the clinical significance of the RAV in patient care and questioned its inclusion in the new guidelines. Concerns have been raised about the practical aspects of measuring the RAV, including considerations of

mechanical ventilation, lung/chest compliance, and diaphragm function. Additionally, questions were posed regarding the methodology for measuring the RAV and the variables involved, such as the type of ventilation, tidal volume, and level of sedation. Some respondents suggested indexing the RAV to tidal volume in mechanically ventilated patients for standardized measurements. Overall, there is a need for further clarification and evidence regarding the clinical utility and measurement methodology of the RAV in assessing abdominal compliance.

Statement 10: *Respiratory abdominal variation test (RAVT) is a non–invasive test assessing RAV during gradual increase in tidal volume (in mechanically ventilated patients) and provides indirect measure of Cab* (overall agreement 91.2%, 32 comments).

Many respondents expressed uncertainty regarding the clinical utility and rationale behind RAVT, questioning the need for a respiratory challenge to the patient and the potential complexities introduced by increasing tidal volume without assessing inspiratory pressure. Concerns have been raised about the accuracy of RAVT in evaluating abdominal compliance, given the various lung factors and chest–wall rigidity that may influence its results. Some respondents suggested that the test should consider changes in lung compliance and the value of positive end–expiratory pressure (PEEP) to provide a more comprehensive assessment. Overall, there is a need for further evidence and clarification regarding the clinical significance and methodology of RAVT in assessing abdominal compliance.

Statement 11: *Positional abdominal variation test (PAVT) is a non–invasive test assessing RAV during gradual changes in head of bed (HOB) [also in spontaneous breathing] and provides indirect measure of Cab* (overall agreement 89.5%, 30 comments).

The respondents expressed a lack of familiarity with PAVT, highlighting the need for more information and clarification regarding its methodology and clinical utility. Concerns have been raised about the variability of measures and potential operator dependency, suggesting that these factors could affect the reliability and clinical relevance of PAVT. Additionally, some respondents questioned the fidelity of PAVT in patients with spontaneous breathing, citing issues such as chest wall resistance and elastance that may impact its accuracy. Despite these concerns, some respondents acknowledged the practicality of PAVT, noting its potential usefulness given that patients typically change their HOB position during hospitalization. Overall, further research and validation are needed to better understand the role of PAVT in assessing abdominal compliance.

Statement 12: *Abdominal pressure variation (APV) is an indirect measure of Cab and can be calculated as ratio of difference between* end-inspiratory IAP (IAP_{ei}) and end-expiratory IAP (IAP_{ee}) and mean IAP (IAPmean) *[IAPei – IAPee)/IAPmean]* (overall agreement 90.5%, 31 comments).

The respondents expressed uncertainty and the need for more information regarding the clinical utility and practical application of APV in patient care. Some respondents questioned the need for verification, validation, and evidence on APV in effective patient management. Concerns have also been raised about operator dependency and the complexity of the formula used to calculate APV,

suggesting a need for further clarification and perhaps simplification. Additionally, there are suggestions for renaming APV to the APV index (APVi) to better reflect the calculation technique. Overall, there is a consensus among respondents that more research and evidence are needed to establish the usefulness and reliability of APV as an indirect measure of abdominal compliance.

Measurement of intrabdominal pressure

Statement 13: *Clinical assessment and estimation of IAP is inaccurate* (overall agreement 65.2%, 31 comments).

The respondents expressed a variety of viewpoints regarding the accuracy and reliability of the clinical assessment and estimation of the IAP. Some respondents emphasized the importance of using standardized measurement techniques, such as bladder pressure measurement, for accurate assessment of IAP. Others highlighted the variability in clinical assessment due to factors such as operator–dependence and individual patient characteristics. There were also comments suggesting that while clinical assessment may provide useful information in some instances, direct measurement of the IAP remains the gold standard for accuracy. Overall, there was a consensus among respondents that the accuracy of clinical assessment and estimation of the IAP varies depending on the method used and the clinical context, with some advocating for more standardized measurement techniques to improve accuracy and reliability.

Statement 14: *The reference standard for intermittent IAP measurement is via the bladder with a maximal instillation volume of 20–25 mL of sterile saline* (overall agreement 87.2%, 44 comments).

There were various opinions among respondents regarding the specific volume of saline instilled. Some respondents suggested that fixing the instillation volume at either 20 or 25 mL rather than within a range would be better. Others have proposed the use of a larger volume, such as 50 mL or even 100 mL, for a more accurate estimation of the IAP. There were also comments expressing concerns about the potential for error with the recommended volume, particularly in specific patient populations such as neonates or anuric patients. Overall, while there was general agreement on the use of bladder pressure measurement as the reference standard for intermittent IAP measurement, there were divergent opinions on the optimal volume of saline instillation, with some advocating for a fixed volume and others suggesting larger volumes for improved accuracy.

Statement 15: *The gastric route can be used as alternative for intermittent IAP measurement with a maximal instillation volume of 50–75 mL of water or nutritional fluid* (overall agreement 70.4%, 71 comments).

Numerous comments indicate uncertainty, lack of experience, and the need for specific guidelines regarding this method. Many respondents expressed doubts about the accuracy and reliability of using the gastric route for IAP measurement. Some mentioned concerns about the feasibility of using high volumes of fluid and the potential for inaccuracies due to gastric contractions, variations in stomach size and tension, and the presence of gastric residue. Others have noted that the stomach is an open cavity, which may

compromise the accuracy of measurements. Overall, while there was agreement that the gastric route could serve as an alternative for IAP measurement, many respondents emphasized the need for further validation, clear guidelines, and evidence supporting its use, as well as consideration of potential limitations and challenges associated with this method.

Statement 16: *IAP should be expressed in mmHg and measured at end–expiration in the supine position after ensuring that abdominal muscle contractions are absent and with the transducer zeroed at the level of the midaxillary line* (overall agreement 91.4%, 34 comments).

Few respondents suggested modifications to the definition to address practical considerations and variations in patient positioning. Many respondents noted that most ICU patients are not in the supine position but rather in a semi–recumbent position with the head of the bed elevated. They suggested modifying the definition to include IAP measurement in the 30–45° head–up position to reflect the clinical reality. Some respondents proposed allowing the presence of minimal muscle contractions in the definition, particularly for mechanically ventilated non–paralyzed patients, to facilitate easier application in clinical practice. Suggestions were made to include additional landmarks, such as the iliac crest or pubis, in the definition for more precise measurement. While the statement specifies “mmHg” as the unit of measurement, some respondents also recommended including “cmH_2O” or including a conversion ratio, especially for low–resource settings where transducers may not be available. There are also suggestions to incorporate dynamic measurement charts and consider body position variations for IAP measurement. Overall, while there was agreement on the

fundamental aspects of expressing and measuring IAP, there were valuable suggestions for refinement to better align with clinical practice and address practical challenges.

Statement 17: *After IAP measurement in the supine position, IAP should also be measured in the "resting" position of the patient, e.g., the normal HOB is at 30–45° position or prone position* (overall agreement 71.9%, 51 comments).

This statement received an overall agreement of 71.9%, and the respondents expressed various concerns. Some have questioned its routine implementation and relevance, suggesting standardization to the HOB or supine position owing to practicality concerns with prone positioning. Others highlighted the need to clarify the importance and implications of measuring the IAP in various positions, with particular scrutiny given to the lack of data supporting the measurement in the prone position. Additionally, there are suggestions to focus on correcting the IAP for patient positioning rather than conducting measurements at multiple positions for each patient. Overall, while there was an acknowledgment of the potential impact of patient position on the IAP, there were reservations about the feasibility and necessity of measuring the IAP in different positions, especially prone positions.

Statement 18: *IAP measurement can also be performed in awake or spontaneously breathing patients* (overall agreement 82.9%, 51 comments).

Among the comments received, respondents generally agreed with the statement but emphasized several caveats. They noted that such measurements should be conducted when the patient is breathing comfortably, with pain under control, and without squeezing or coughing. Some expressed concerns about the reliability and standardization of measurements in awake patients, highlighting potential challenges such as interpreting results and accounting for variations in muscle tone. Additionally, there were suggestions to compare measurements in awake patients with baseline assessments in nonspontaneously breathing states to provide context for the values obtained. While the feasibility of measuring the IAP in awake patients has been acknowledged, there have also been reservations about its accuracy and interpretability, necessitating careful consideration and further validation.

Statement 19: *Normal IAP is approximately 5–7 mmHg in critically ill adults* (44 comments). *Suggestion to modify as follows: IAP is approximately 5–7 mmHg in healthy adults* (overall agreement 82.1%, 44 comments).

Among the comments received, there were queries about the evidence supporting this assertion and concerns about whether the term "healthy" includes individuals with conditions such as obesity or pregnancy. The respondents emphasized the absence of a sharp cutoff between normal and pathological values, suggesting a broader range for physiological IAP. Some researchers have proposed modifications to the statement, advocating for a range of 0–5 mmHg in healthy adults and emphasizing the impact of factors such as body mass index (BMI) and bodybuilding on the IAP. There were also considerations about variations in IAP on the basis of patient population and comorbidities, highlighting the need for clarity and further evidence to support the suggested modification

Statement 20: *Normal IAP is approximately 10 mmHg in critically ill adults* (overall agreement 77%, 62 comments).

There are queries about the evidence supporting this assertion and concerns about defining a single value as "normal" for critically ill patients, given the variability in disease and patient characteristics. The respondents emphasized the need to consider a range rather than a specific value, with suggestions ranging from 7 to 12 mmHg. Concerns have been raised about the broad spectrum of critically ill patients and the need for uniformity in defining normal IAP across different clinical scenarios. Some respondents advocated considering factors such as BMI when determining IAP levels. Overall, there is a consensus that defining IAH and ACS should be the primary focus rather than specifying a single value for a normal IAP in critically ill adults.

Statement 21: *The normal IAP differs regarding the patient population and anthropometry and can be non–pathologically increased 10–15 mmHg in obese patients, pregnancy, etc* (overall agreement 92%, 31 comments).

Few comments expressed the need for evidence to support this claim and advocated for further studies to verify these assertions, particularly through anthropometric studies. Some respondents emphasized the importance of considering the physiological impact of elevated IAP rather than simply labeling it as normal. Concerns have been raised about the specific range proposed, with suggestions to lower the upper limit or to individualize interpretations on the basis of the clinical context. Additionally, while certain conditions, such as obesity, may lead to elevated IAP, they do not necessarily make it normal and may instead indicate IAH. Overall,

while there was general agreement about the variability of IAP across different patient populations, there were reservations about defining a precise range that needed more robust evidence to support it.

Statement 22: *Different techniques exist to perform continuous IAP monitoring (e.g., gastric, bladder, direct). A gold standard yet needs to be identified* (overall agreement 89.8%, 37 comments).

There are few comments that underscore the ongoing debate surrounding the identification of a gold standard technique. While many participants advocated for the bladder method, citing its widespread clinical use and accessibility, others suggested that no gold standard has yet been definitively identified. Some highlighted the complexities and limitations of alternative techniques, such as gastric monitoring in patients receiving continuous tube feeds and direct methods being too invasive. Despite these divergent opinions, there is a consensus on the need for further research and standardization efforts to establish a gold standard technique for continuous IAP monitoring, reflecting the ongoing evolution and refinement of critical care practices in this domain.

Statement 23: *Continuous IAP can be used to keep track of changes in IAP during treatment* (overall agreement 91.9%, 29 comments).

Amidst this consensus, there were few comments expressing diverse considerations and reservations. Some emphasized the significance of trends over singular measurements, advocating for metrics such as time above threshold and area under the curve

for patient monitoring. Bladder pressure measurement is reliable, and it is the present gold standard; moreover, it is inexpensive and well–studied. Others called for standardizing continuous IAP monitoring as part of clinical practice, albeit with concerns raised about potential variations due to changes in patient position. Practical challenges, including maintaining a steady-state condition, patient selection, and body position, were also noted. While some support the routine use of continuous monitoring, others question its necessity and feasibility in clinical settings. Furthermore, there have been discussions about the superiority of individual methods of continuous monitoring over each other and over intermittent measurements, alongside concerns about the reliability (the need for recalibration in the case of a single sensing balloon as air may diffuse), costs and longevity of monitoring systems. In addition, the FDA has already approved several new technologies as continuous IAP measurement devices (TraumaGuard, Serenno). These diverse perspectives underscore the need for further research, standardization efforts, and careful consideration of implementation strategies to optimize the use of continuous IAP monitoring in clinical practice.

Definitions

Statement 24: *IAH is defined by a sustained or repeated pathological elevation in IAP >12 mmHg* (overall agreement 90.9%, 25 comments).

There are a few suggestions for revising the threshold of the IAP to greater than 15 mmHg, emphasizing the importance of correct measurement techniques and providing clarity over what constitutes repeated or sustained elevation. Concerns also arise regarding the clinical implications, with some proposing tailored assessments on the basis of individual patient characteristics and end–organ effects, particularly in obese individuals, where the relationship between IAH and other clinical parameters may differ. Furthermore, discrepancies emerge regarding the relationship between IAH and other clinical parameters in children (needing lower thresholds), warranting further elucidation and refinement of the definition to ensure clinical relevance and accuracy across diverse patient populations. Therefore, this definition is probably valid only in nonobese adults.

Statement 25: *ACS is an all or nothing phenomenon and defined as a sustained IAP >20mmHg (with or without an APP <60mmHg) that is associated with new organ dysfunction/failure* (overall agreement 89.1%, 46 comments).

Some respondents challenged the notion of ACS as an "all or nothing" phenomenon, advocating for a more nuanced approach given the dynamic nature of IAP and its impact on organ function rather than a single value. Others emphasize the importance of focusing on new or aggravating organ dysfunction rather than a specific pressure threshold, suggesting a clinical definition with easy reproducibility in low- and middle-income countries (LMICs). The inclusion of the app in the definition raises questions about its relevance and the need for further study, especially regarding its role as an endpoint for management. The 2013 WSACS guidelines did not support the use of apps. More studies are needed to validate the APP as a clinically relevant resuscitation endpoint, and the

APP should probably also be personalized or individualized on the basis of comorbidities and patient history. Additionally, there are proposals to modify the definition to capture a broader spectrum of patients, considering the continuum from IAH to ACS. These comments highlight the complexity and ongoing debate surrounding the definition of ACS and underscore the need for continued research and consensus-building in this area.

Statement 26: *Organ dysfunction/failure is assessed by (a daily) SOFA or equivalent scoring system (qSOFA); organ failure is defined as a SOFA organ system sub–score of >2* (overall agreement 87.5%, 46 comments).

Many respondents argued against the inclusion of the quick SOFA (qSOFA) score because of its low specificity and limited applicability in critically ill patients. Instead, there was a call to focus on organ failure rather than specific scoring systems, emphasizing clinical judgment and a combination of criteria such as hemodynamics, respiratory issues (including compliance), and signs of poor perfusion in the kidneys and gastrointestinal system. Some have proposed expanding the assessment to include parameters such as lactate levels, cardiovascular instability, respiratory mechanics, and gastrointestinal dysfunction scores to better capture the complexity of organ dysfunction in ACS. Others advocate for a more nuanced approach, considering individual organ dysfunctions and utilizing specific criteria such as the RIFLE to assess renal function. These comments highlight the need for a comprehensive and adaptable approach to assess organ dysfunction/failure in ACS, taking into account the multifactorial nature of the condition and the diverse clinical presentations of affected patients.

Statement 27: *Sustained increase in IAP is defined as a pathological value during a minimum of three standardized measurements that are performed 1–2 hours apart for ACS and 4–6 hours apart for IAH* (overall agreement 89%, 38 comments).

The respondents expressed several concerns and suggestions regarding this definition. First, some argue that the time interval between measurements should be shorter, especially in cases of abdominal compartment syndrome (ACS), where immediate action is necessary. Instead of waiting for several hours, it was suggested that two measurements taken within an hour apart could be more appropriate to prompt timely intervention. Additionally, there was a call for continuous monitoring of the IAP, especially in critically ill patients, as even a single elevated reading could indicate significant pathology. Continuous monitoring could provide a more comprehensive understanding of IAP dynamics and facilitate prompt management. Furthermore, the timing of the differentiation between ACS and IAH is unclear. It was suggested that the timeframe for defining a sustained increase in IAP should be consistent for both conditions. Finally, the definition should consider the clinical context, especially if active interventions are implemented to manage rising IAP. In such cases, more frequent measurements may be warranted to guide timely interventions and prevent complications. In summary, respondents advocate for a more flexible and clinically relevant definition of a sustained increase in IAP, considering the urgency of intervention, the need for continuous monitoring, and the clinical context of individual patients.

Statement 28: *IAH is a continuum and graded as follows: Grade I, IAP 12–15 mmHg, Grade II, IAP 16–20 mmHg, Grade III, IAP 21–25 mmHg, and Grade IV, IAP > 25 mmHg* (overall agreement 91.4%, 32 comments).

The current grading system for intra-abdominal hypertension (IAH) faces criticism and suggestions for improvement from a few respondents. One prevalent critique is that the current grading system lacks clinical relevance and meaningful distinctions between grades. Some suggest simplifying the grading system into two categories: one for IAP levels between 12–20 mmHg and another for IAP levels exceeding 20 mmHg. This more straightforward approach could facilitate clearer clinical decision–making, especially concerning the need for interventions and the risk of developing ACS. Furthermore, there was a proposal to merge grades III and IV, as the differences between them are minimal in terms of clinical implications and outcomes. Combining these grades could streamline the grading system and reduce unnecessary complexity. Some respondents questioned the necessity of Grade I restraint in the grading system, suggesting that it may not have significant clinical implications or clear distinctions from Grade II restraint. Additionally, concerns are raised about the lack of outcome correlates for the current grading system, highlighting the need for more research. Simplification, merging redundant categories, and establishing clearer clinical implications are key considerations for enhancing the current grading system.

Statement 29: *Primary IAH or ACS is a condition associated with injury or disease in the abdominopelvic region that frequently requires early surgical or interventional radiological intervention* (overall agreement 86.8%, 23 comments).

Some respondents made several pertinent comments highlighting the need for clarification and refinement. The respondents emphasized the importance of exploring medical interventions as the initial approach, particularly before considering surgical or interventional radiological interventions. This includes interventions such as diuresis and fecal dis–impaction, which can be effective in managing certain cases of IAH. There's a call for clarity regarding the types of interventions encompassed by the term "interventional radiological intervention." The respondents suggested including examples such as ascites drainage, which can be performed not only by interventional radiologists but also by other medical practitioners. The respondents stressed the importance of differentiating between interventions required for IAH and those specifically needed for ACS. While ACS may necessitate surgical intervention, the same may not apply to all cases of IAH. Therefore, there is a need to reduce aggressive surgical exploration and prophylaxis for ACS in patients with IAH, considering the associated morbidity of open abdomen procedures. Some respondents advocated for nuanced wording in the statement, suggesting that phrases such as "may require" or "strongly consider" instead of definitive language. This nuanced approach acknowledges the varied clinical scenarios and individual patient factors that may influence the need for intervention. These findings underscore the importance of exploring nonsurgical treatment options before invasive interventions are considered. This aligns with the principle of conservative management and emphasizes the need for a tailored approach to patient care.

Statement 30: *Secondary IAH or ACS refers to conditions that do not originate from the abdominopelvic region* (overall agreement 89.4%, 17 comments).

Few respondents expressed concerns about the potential need for prompt surgical intervention despite the condition being classified as secondary IAH or ACS. Some expressed uncertainty about the distinction between primary and secondary IAH, particularly in cases where the condition may originate from the abdominopelvic region as a complication of previous events. There are suggestions to link the definition of secondary ACS with associated requirements for massive resuscitation and to consider more objective criteria for its diagnosis. Additionally, there are queries about whether conditions originating from the retroperitoneum should be classified as primary or secondary ACS, highlighting the need for further clarification. Finally, distinguishing between primary and secondary IAH can be challenging, especially in cases where there is pain and high tension in the abdominal muscles.

Statement 31: *Recurrent IAH or ACS refers to the condition in which ACS redevelops following previous surgical or medical treatment of primary or secondary ACS* (overall agreement 93.8%, 13 comments).

Some suggestions were to use "despite" instead of "following" to emphasize the persistence of ACS despite prior treatment. There were also suggestions to maintain reference to both IAH and ACS in the statement, as it initially referred to both but later mentioned only ACS. Concerns have been raised about the significance of recurrent IAH, particularly considering that many patients still experience IAH after decompression for ACS. Additionally, it was noted that the definition should include documentation of the

resolution of prior ACS for clarity. Some respondents proposed the use of the term "tertiary ACS" to describe recurrent IAH or ACS, emphasizing that it can occur even after primary or secondary ACS treatment. There are also suggestions to define the time interval between the occurrence of IAH/ACS and previous surgery to provide clearer criteria for diagnosis. Furthermore, it was suggested that IAP measurements and interpretations be reconsidered after damage control surgery and/or an open abdomen.

Statement 32: *The four distinct IAH categories are defined as medical, surgical, trauma or burns* (overall agreement 91.6%, 33 comments).

The relevant comments concerned the clinical usefulness of this classification, noting overlap between categories and the lack of clear boundaries. Suggestions were made to consider alternative classifications on the basis of factors such as wall compliance, intra– or extraluminal contents, fluid resuscitation, or capillary leakage. Questions were also raised about the inclusion of specific conditions, such as pancreatitis and pregnancy, as well as iatrogenic causes. Some respondents proposed simplifying the classification to primary and secondary IAH instead of delineating individual categories. Concerns were expressed about the potential confusion caused by overlapping categories, such as trauma and burns being considered surgical issues. Additionally, the classification may not adequately capture the diverse etiologies of IAH. Overall, the respondents questioned the relevance and clarity of the defined categories and suggested reconsidering the classification scheme to better reflect the clinical reality.

Statement 33: *Localized IAH and ACS is defined as a local increase in IAP that does not lead to a systemic elevation (e.g., pelvic trauma, liver or spleen trauma)* (overall agreement 87.5%, 30 comments).

Some respondents raised concerns about the conceptualization and measurement of localized IAH. Few studies have argued that a localized increase in pressure that does not manifest as a systemic elevation should not be classified as IAH. Others suggested revising the term "organ-specific IAH" to better reflect the anatomical location, e.g., local kidney compartment syndrome, local hepatic compartment syndrome, etc. Concerns were expressed about the need for more clarity in defining localized ACS, as ACS typically involves the entire peritoneal cavity and results in organ dysfunction. The respondents also questioned the clinical usefulness of the concept, noting the difficulty in measuring localized pressures and confirming their systemic effects. Overall, there was uncertainty about the definition and measurement of localized IAH and ACS, with some suggesting the need for more objective criteria to define these conditions.

Statement 34: *IAH duration can be chronic, acute, subacute or hyperacute* (overall agreement 88.2%, 25 comments).

Few respondents expressed concerns about the complexity and ambiguity of the terminology, suggesting that acute and hyperacute categories overlap and may be confusing. Some have suggested simplifying the classification to acute and chronic, whereas others have questioned the evidence supporting the proposed categories. Concerns have been raised about the lack of clear definitions and

the potential confusion these terms may introduce in clinical practice. Overall, while some respondents acknowledged the validity of the classification, they also considered it arbitrary and recommended clearer definitions or simplifications of the terminology.

Statement 35: *Chronic IAH is defined as IAH that lasts for months or years (e.g., ovarian tumor, ascites, pregnancy)* (overall agreement 90.8%, 25 comments).

The respondents raised concerns about the clinical relevance of the term "chronic IAH" and suggested excluding certain conditions, such as pregnancy and obesity, from this definition. Some suggested specifying the duration more precisely, with suggestions ranging from weeks to months. Concerns have also been raised about whether chronic IAH leads to ACS and whether the term should be abandoned altogether. Overall, the respondents highlighted the need for clarity and specificity when the term "chronic IAH" was introduced into the classification.

Statement 36: *Acute IAH is defined as IAH that develops within hours (e.g., ruptured AAA)* (overall agreement 93.3%, 14 comments).

Some respondents raised concerns about the specificity of the timeframe, suggesting that acute IAH can develop within minutes or up to 48 hours. Some questioned the clinical significance of differentiating between forms of IAH, expressing worries that it may overcomplicate matters. Suggestions were made to fold acute IAH into the concept of ACS and to consider including conditions such

as severe burns in the definition. Clarification of the timing and clinical relevance of defining acute IAH is recommended for further consideration.

Statement 37: *Subacute IAH is defined as IAH that develops within days (e.g., fluid overload and capillary leak)* (overall agreement 94.1%, 18 comments).

Some respondents questioned the specificity and clinical relevance of the term "subacute," suggesting that it might be vague. There are suggestions to reconsider whether it makes sense to separate IAH into subacute categories, with some proposing to include subacute IAH within the acute category or ACS. Recommendations were made to define the timeframe more precisely, such as within 24 hours or a minimum number of days, to enhance clarity and clinical applicability.

Statement 38: *Hyperacute IAH is defined as IAH that only lasts for second or minutes (e.g., coughing, sneezing)* (overall agreement 82.6%, 56 comments).

Several respondents expressed concerns about the clinical relevance and utility of this term. They questioned the need to define hyperacute IAH, which describes physiological events such as coughing or sneezing, which are not pathological conditions. Some have suggested that this definition might add confusion and unnecessary complexity to the concept of IAH. Recommendations

were made to consider alternative terms such as "transient" or "physiological," and some suggested excluding hyperacute IAH altogether owing to its limited clinical importance.

Management of IAH/ACS

Statement 39: *For further fine–tuning and classification of IAH/ACS four questions need to be answered. 1. What is the duration of IAH/ACS? 2. Is an intra–abdominal problem responsible for the IAH/ACS? 3. What is the aetiology of the IAH/ACS? 4. Is there a local compartment syndrome?* (overall agreement 92.7%, 27 comments).

The respondents suggested that offering a severity grading system would be beneficial for better classification. Some participants noted that questions 2 and 3 are redundant and essentially ask the same thing and suggested combining them into one question to avoid redundancy. There were doubts expressed about the relevance of Question 4, questioning its importance in the fine-tuning process. Further clarification was requested for the term "local" in Question 4 to better understand its significance. Finally, some respondents proposed adding additional questions, such as assessing the kinetics or consequences of the condition and whether any previous therapies for IAH/ACS had failed. Overall, while there was general agreement on the need for further fine-tuning and classification, there were suggestions for refinement and clarification of the proposed questions.

Statement 40: *The open abdomen is one that requires a temporary abdominal closure (TAC) due to the skin and fascia not being closed after laparotomy* (overall agreement 92.1%, 17 comments).

Some relevant comments from respondents suggest modifications to the definition. Few respondents noted that while the fascia may remain open, the skin can sometimes be closed. It was suggested that the definition should focus more on the fascia rather than the skin closure. Notably, the abdomen can be left open to heal by secondary intention, indicating that closure may not always be necessary. There are suggestions to emphasize the temporary nature of open abdomen management, as it is often a TAC. The inclusion criteria for patients who were reexplored and for whom the abdomen remained open due to the need for reoperation, such as mesenteric ischemia, were highlighted for consideration in the definition. In summary, while there was agreement with the essence of the statement, there were suggestions to refine the definition to better reflect the clinical nuances and variations in practice regarding open abdomen management.

Statement 41: *Lateralization of the abdominal wall is the phenomenon where the musculature and fascia of the abdominal wall, most exemplified by the rectus abdominus muscles and their enveloping fascia, move laterally away from the midline with time* (overall agreement 93.9%, 16 comments).

While there was a high level of agreement with the statement, few respondents suggested modifying the definition. One comment suggested that the language used in the statement was unclear, indicating a need for clarification. Additionally, some respondents

noted that the phenomenon described may be more commonly known as "loss of domain" rather than lateralization of the abdominal wall.

Statement 42: *When left open with a TAC, the open abdomen should be closed as soon as possible (usually within one week)* (overall agreement, 87.3%; 29 comments).

Several comments suggested modifying the definition to include a shorter timeframe for closing the open abdomen with a TAC. Suggestions include closing within 72 hours for optimal results, with some expressing doubts about waiting for a whole week. Others emphasize the importance of considering clinical conditions and the resolution of the underlying pathology in determining the timing of closure. Additionally, there is a suggestion to avoid specifying a particular time frame and instead focus on closure when clinically feasible, preferably within a week.

Statement 43: *Medical management is defined as a nonsurgical intervention with the purpose to lower increased IAP and consists of five treatment options: improvement of Cab, decrease of intra–abdominal volume, decrease of intra–luminal volume, fluid management, organ support* (overall agreement 95.4%, 13 comments).

Some relevant comments focused on enumerating treatment options, and the definition should focus on nonsurgical interventions aimed at lowering increased IAP. Specifically, interventions such as improvements in abdominal compliance, reductions in intra-

abdominal volume, management of intraluminal volume, fluid optimization, and organ support should be included. Additionally, the term "fluid management" should be clarified to encompass concepts such as diuresis. Moreover, additional interventions, such as pain control, changes in body position, bowel enemas, and muscle relaxation, should be incorporated. Furthermore, including medical management specifically for ACS is recommended, as there is no evidence for treating IAH without ACS. Finally, the relationship between intraluminal volume and intraabdominal volume should be addressed, as they are interconnected because of the positioning of hollow viscera within the abdominal cavity.
